# Engagement With Meditation Apps: Cross-Sectional Survey of Use and Associations

**DOI:** 10.2196/71960

**Published:** 2026-02-02

**Authors:** Julia Adams, Jonathan Davies, Prai Wattanatakulchat, Julieta Galante, Felicity Miller, Simon D'Alfonso, Nicholas T Van Dam

**Affiliations:** 1 Contemplative Studies Centre Faculty of Medicine, Dentistry and Health Sciences University of Melbourne Melbourne Australia; 2 School of Computing and Information Systems University of Melbourne Melbourne Australia

**Keywords:** meditation, mindfulness, engagement, app, meditation apps, digital mental health intervention, behavior change

## Abstract

**Background:**

Meditation apps are increasingly popular, yet there is limited understanding of how much users actually engage with them. While meditation apps show promise for supporting mental health, engagement in real-world settings appears to be notably low. The patterns of app use and the factors that influence usage remain relatively unclear.

**Objective:**

This study aims to examine the extent of meditation app use and the factors associated with user engagement.

**Methods:**

We conducted a cross-sectional survey of 536 recent meditation app users across 5 English-speaking countries. Engagement data were collected via self-report and app-verified screenshots. Assessed factors included user characteristics (age, education, income, sex, country, personality, self-efficacy, readiness and expectations for change, self-compassion, and quality of life), mental health (distress, well-being, life satisfaction, anxiety, depression, support, and stress), and app-related elements (therapeutic alliance, appeal, functionality, aesthetics, information, quality, and perceived impact). The 4 outcome variables representing engagement were app-verified minutes, self-reported minutes, app-verified minutes per year (adjusted for app download date), and self-reported minutes per year (adjusted for app download date). Associations between app use and variables of interest were examined using correlations. Factors with significant associations were then included in multivariable regression models to identify those most strongly associated with engagement.

**Results:**

Age (ρ=0.13-0.15, PP^FDR^, where FDR is false discovery rate), expectations for sleep (ρ=0.12-0.33, P^FDR^<.05), and expectations for thriving (ρ=0.12-0.18, P^FDR^<.05) were associated with all outcome measures except adjusted objective minutes. Readiness to change was associated with all outcome measures (ρ=0.24-0.33, P^FDR^<.05). Among app factors, appeal (ρ=0.18-0.23, P^FDR^<.05) and perceived impact (ρ=0.23-0.32, P^FDR^<.05) were associated with all outcome measures except adjusted self-report minutes, while perceived quality (r=0.28-0.51, P^FDR^<.05) was associated with all outcome measures. Robust linear regressions showed that greater readiness to change (β=0.005-0.026, *P*=.006-.02), higher education level (β=0.029-0.540, *P*<.001), and higher openness (β=0.004-0.010, *P*=.008-.03) were associated with increased engagement. Additionally, greater expectations for sleep (β=0.004-0.009, *P*=.02-.04), greater expectation match (β=0.023, *P*=.03), and higher perceived app quality (β=0.008-0.042, *P*=.001-.01) were uniquely associated with increased engagement.

**Conclusions:**

Most individuals who download meditation apps engage minimally. Our findings suggest that users who are more educated, open to new experiences, and hold strong beliefs in the effectiveness of meditation apps are more likely to use them regularly. Longitudinal studies are needed to examine patterns of use and strengthen causal inferences.

## Introduction

### Background

Around 1 billion people globally live with a mental health disorder [1], creating a demand that exceeds available resources. As the rise of technology has coincided with increasing strain on mental health systems, digital mental health interventions have gained popularity due to their accessibility [2,3]. Fully automated versions of these interventions may reduce reliance on limited human resources. However, engagement remains a challenge, with fewer than 20% of users continuing beyond 7 days [4].

Meditation apps are among the most common digital mental health tools [4,5]. Meditation encompasses a wide range of techniques across different traditions and religions and typically involves emotional and attentional regulation [6]. Mindfulness meditation, for example, emphasizes nonjudgmental awareness of the present moment [7]. Among regular meditators, most have used a meditation app [8]. While global rates of meditation remain unknown, 70 million people had downloaded Headspace by 2022 [9]. Thus, it is likely that a very large proportion of the population has tried meditation through an app. Given the high accessibility of meditation apps among those facing barriers to mental and physical health care, it is important to examine the practical limitations of such programs, with engagement being a key shortfall in app-based behavior change [10,11].

Most current information on meditation app use comes from clinical trials, which are not representative of real-world use. While randomized controlled trials of meditation apps show small- to medium-sized effects [12], real-world estimates suggest exceedingly high discontinuation rates [4]. As behavioral interventions are only effective if used, this presents a major challenge for apps [4]. For digital offerings to be truly useful, a better understanding of the factors associated with sustained use is essential. This study examines engagement rates and identifies who is most likely to engage with meditation apps.

### Mindfulness-Based Programs and Meditation Apps

While a limited number of meditation apps have shown some efficacy for mental health outcomes [12], they should be considered separately from the established evidence base for mindfulness-based programs (MBPs) [13]. MBPs are arguably the most popular form of meditation training in clinical and academic settings, likely due to their strong evidence base [14,15]. By contrast, most popular meditation apps depart from the guided, intensive structure of MBPs [16]. Only 4% of popular apps provide evidence of their benefits [17]. Even where apps show potential efficacy, recent reviews highlight engagement as a major limitation to intervention effectiveness [12,18-20].

### The Digital Transition

Digital health interventions generally face engagement issues, which likely reduce their benefits [4]. In nonpharmacological interventions, adherence is linked to outcomes [21], yet it tends to be worse in digital formats than in face-to-face interventions [22]. For behavior change apps (and apps more broadly), discontinuation occurs in 40%-60% of users [11]. In naturalistic settings, 21%-88% of users engage with an app at least once, but only 0.5%-28% sustain engagement (eg, completing all assigned modules or continuing use beyond 6 weeks [23]). Engagement decreases when digital interventions lack interactive human or human-like support [24], posing challenges for fully automated meditation apps. Clarifying who engages meaningfully with meditation apps is therefore important, given the link between adherence and outcomes.

### Attrition, Adherence, and Engagement

Engagement refers to the extent of intervention use, including the amount, frequency, duration, and depth of use [25]. Attrition and adherence are related terms that describe levels of (dis)engagement in research studies. Attrition refers to discontinuation or dropout from the intervention program or from research data provision during a study [11]. For MBPs, attrition is around 19% [26], whereas app-based interventions show an average attrition of 42% in studies lasting 10 days to 12 weeks [12]. Real-world estimates for meditation apps indicate disengagement rates as high as 94% within the first 2 weeks [4].

Adherence refers to the extent to which an individual follows a prescribed treatment or intervention [21]. As no clear guideline exists for the amount of practice required to achieve an effect in mindfulness or meditation [16,27], adherence can only be considered in relation to recommended practice amounts (see example in [28]). In meditation training, prescribed engagement time ranges from as little as 35 minutes [28] to 3 hours per week in the widely used Mindfulness-Based Stress Reduction program [7]. By contrast, many apps recommend as little as 5 minutes per day or provide no clear guidance regarding minimum practice length, session duration, or overall time commitment needed to establish a practice [29]. Given the limited knowledge about dose-response relationships in meditation [16,29] and the tendency for most people to discontinue practice relatively early [4], engagement serves as a useful proxy for understanding who practices, what type of practice they follow, and why.

### Why Do People (Dis)Engage With Meditation and Apps?

#### Overview

Understanding engagement in meditation apps requires consideration of various behavior change and persuasive systems design frameworks. Behavior change frameworks—including habit formation theory, social cognitive theory, the theory of planned behavior, and the transtheoretical model of change—suggest that user characteristics such as expectations, motivation, readiness to change, consistency of use, and self-efficacy influence engagement with behavioral interventions [30-33]. The persuasive systems design framework highlights app design features that shape engagement and therapeutic alliance [34].

Habit formation theory emphasizes reward and associative cues as central to establishing habits, with positive outcomes reinforcing continued engagement [33]. Context can shape how rewards are perceived, influencing whether a habit is formed [35]. The theory of planned behavior further posits that perspective and context guide behavior [30,36]. Broad factors such as sociodemographics, mental health, and personality also influence engagement [30]. Expectations are shaped by attitudes and norms, with positive expectations and attitudes predicting greater meditation app engagement [36]. The transtheoretical model outlines stages of change, with later stages—more closely aligned with commitment to action—linked to more sustained behavior change [32]. Readiness to change reflects an individual’s stage of change and is associated with successful maintenance of behavior change [37]. The Sussex Meditation Model identifies preintention, preparation, action, and maintenance stages as relevant to establishing a meditation practice [38,39]. Persuasive systems design, which examines how digital interventions can be structured to influence user behavior, highlights app features that enhance engagement, such as reminders and personalization [34]. Drawing on these frameworks, factors relevant to engagement in behavior change, meditation, or app use were categorized into user-related factors (sociodemographics, personal/user characteristics, and mental health factors) and app-related factors.

#### Sociodemographic Factors

Sociodemographic factors associated with disengagement from meditation include lower levels of education [40]; however, men, people with less education, and those with poorer health are less likely to begin meditating [41]. Meditators are also more likely to be wealthier than nonmeditators [41]. In online and app-based meditation, older age, positive expectations, and intrinsic motivation are associated with greater engagement [42,43].

#### Personal/User Factors

Personality factors have also been shown to influence engagement with meditation apps. Conscientiousness has been associated with meditation in general [44]. Openness predicts meditation practice outside formal program training, reflecting the “in-the-wild” context of app use [45].

Behavior change factors may also influence engagement with meditation apps. Self-efficacy and readiness to change have been linked to successful habit formation [46]. A higher intention to practice is associated with greater engagement [46]. Intrinsic motivation moderates behavior change success across demographic groups [47] and is crucial for making initial behavior change choices. Self-compassion and self-efficacy have also been found to be positively associated with engagement in behavior change [47,48].

Expectations for program efficacy can also influence behavior change. Positive experiences that meet expectations can facilitate ongoing engagement. Conversely, engagement may decline when a program or behavior does not deliver the anticipated positive outcomes [49]. Experiences of progress enhance engagement in both behavior change apps and meditation apps [50,51]. Positive expectations also predict higher engagement with digital meditation resources [42].

#### Mental Health Factors

Health characteristics are also important for engagement. People may be motivated by physical or mental health issues, but these same issues can also act as barriers [43]. This paradox can be explained by the desire to address a problem that simultaneously hinders the ability to engage in practice. Additionally, limited perceived gains may lead to early discontinuation. Barriers to mental or physical health care, which can impact quality of life, may further motivate meditation app use to address unmet health needs [11,41]. Although meditation use among individuals with mental health problems is common, depression is associated with low adherence to behavior modification recommendations in clinical populations [52,53]. The very symptoms people seek to address—such as amotivation, distressing thoughts, and irritability—can also complicate their efforts. Meditation apps may be moderately effective for depression, anxiety, and stress [12,19,50], potentially fostering an experience of progress. However, a minimal level of engagement is necessary to achieve efficacy [4,27]. Consequently, failure to achieve expected outcomes may lead to decreased engagement.

#### App Factors

The user’s relationship with the app is also relevant. Therapeutic alliance—the collaborative relationship between the user and the app—predicts engagement with mental health apps [53]. Ease of use, the ability to personalize settings, reminders, progress tracking, and positive perceptions of the app also predict higher engagement with mental health apps, though these factors have not been extensively examined in meditation apps [42,53,54]. Usability (ie, the app’s functionality) was identified as a key factor related to engagement in a systematic review of mental health apps [55].

### This Study

Previous literature highlights factors that may be associated with meditation app use. In a cross-sectional survey capturing demographics, retrospective reports of app use, mental health factors, and perspectives on apps, we aimed to examine engagement rates and identify factors significantly associated with engagement.

This study focused on several preregistered questions:

1. To what extent are *user-related factors*—including sociodemographic characteristics, spirituality, personality, self-efficacy, self-regulation, motivation, expectations, self-compassion, mental health care status, and psychological distress—associated with mindfulness *app engagement*?
2. To what extent are *user-app relationship factors*—including therapeutic alliance, agreement on tasks and goals, and perceived app empathy and expertise—associated with mindfulness app *engagement*?
3. To what extent are *app-related factors*—including appeal, functionality, aesthetics, information quality, quantity, and credibility, customization, accessibility, and usability—associated with mindfulness app *engagement*?

Additional questions included in the preregistration are not addressed in this paper.

## Methods

### Deviations From Preregistration

For clarification, we have changed the term “mindfulness apps” to “meditation apps” to capture a broader range of relevant practices. Mindfulness can, but does not necessarily, entail meditation and is variably represented as a capacity, skill, or technique. Meditation, by contrast, encompasses a broad array of spiritual and secular practices that use techniques such as focusing on an object, experience, image, or idea [56].

Deviations from the preregistration included the following: (1) focusing on 4 definitions of minutes as the primary outcome and omitting the second preregistered outcome variable—regular practice hours—for simplicity. Regular practice hours were not included because their calculation combined multiple variables and, therefore, could be subject to estimation error. The 4 variations of the outcome variable were included to capture the complexity of user behavior. (2) We did not report 95% odds ratios, as continuous outcomes were used. (3) The final sample size was substantially reduced to 536 from the target of 1000 due to a smaller-than-anticipated eligible pool. This reduction decreased statistical power, although it still allowed adequate power to detect small effects. The reduced pool also led to a fourth deviation. (4) Recruitment was extended to Australia, Canada, the United Kingdom, and New Zealand. An additional deviation involved (5) not analyzing motivation for use, as this information was captured in open-text responses and could not be used in this quantitative analysis.

### Ethical Considerations

This study was conducted in accordance with ethical guidelines and was approved by the Office of Research Ethics and Integrity at the University of Melbourne (approval number 2025-23969-62994-8).

Participants provided informed consent to participate in the study via the Qualtrics survey (Qualtrics International Inc). Consent was obtained within the survey, which also included a downloadable copy of the plain language statement. The plain language statement is available in Section S1 in Multimedia Appendix 1. Provision of informed consent included acknowledgment of the right to withdraw at any time without providing an explanation. Participants also consented to secondary analyses. Survey questions were coded so that participants could not proceed without providing consent. All included responses were double-checked to ensure consent had been given.

Participants were compensated Aus $0.30-0.50 (US $0.20-0.33) for completing the screening survey (mean duration 1 minute 49 seconds) and Aus $6-8 (US $3.96-5.29) for completing the follow-up survey (mean duration 22 minutes 57 seconds), averaging Aus $20.59 (US $13.60) per hour. Survey compensation varied slightly based on median completion time; compensation was occasionally increased to better approximate the proposed hourly rate if the median completion time indicated the study took longer than expected.

### Privacy and Confidentiality

Where possible, identifying information was removed from the dataset. Any copies of datasets containing identifying information were stored securely in accordance with relevant privacy guidelines and encrypted using Transport Layer Security (also known as HTTPS).

### Study Design

#### Overview

This was a cross-sectional analysis of data collected from participants.

#### Procedure

Participants were recruited via Prolific (Prolific Academic Ltd) to complete a survey hosted on Qualtrics. The survey was accessible to potential participants in the United States, the United Kingdom, Canada, Australia, and New Zealand between August 1 and October 6, 2023. Participants were invited to complete a prescreening survey, and eligible individuals were sent the full survey within 1-2 days. Surveys were completed online using a laptop or mobile device. Participants were asked to upload a screenshot of their app use statistics, which provided information such as minutes, days, sessions, streaks, and the original date of download, depending on the app.

#### App Selection

We collected engagement information for popular meditation apps listed on the iOS (Apple Inc) and Android (Google LLC/Alphabet Inc) app stores (see Section S2 in Multimedia Appendix 1). Participants using apps in which meditation—including mindfulness meditation—was the primary intended function were included, based on app descriptions, marketing, and in-app features.

Participants using any app could complete the prescreening survey. Two (JA and JD) researchers assessed whether the app (1) prominently promoted itself as a mindfulness meditation tool and (2) provided techniques to practice mindfulness or another form of meditation. Meditation or mindfulness could not be a secondary component. We did not evaluate app content in relation to any specific definition. We adopted this approach because meditation apps do not offer a single type of meditation, nor do mindfulness apps (eg, Headspace) necessarily adhere to the MBP definition of meditation. Apps were excluded if they focused exclusively on fitness/exercise, employee well-being, cognitive behavioral therapy, or other mental health interventions. All included apps were fully automated (ie, without human support).

#### Participants

Inclusion criteria required participants to have used an eligible meditation app within the past 180 days; be fluent in English; and reside in Australia, Canada, New Zealand, the United Kingdom, or the United States. Exclusion criteria included failure to provide evidence of app use or use of an app in which meditation was not the primary focus.

A total of 6137 prescreening surveys were completed. We excluded 5307 responses: 4343 (70.77%) were unable to demonstrate access to an app, 316 (5.15%) had not used the app in the past 180 days, 319 (5.20%) had downloaded an ineligible app, and 329 (5.36%) self-reported zero use. Of the remaining surveys, 800 (13.04%) met the inclusion criteria.

Of the 800 participants invited to the survey, 677 (84.6%) completed it. Among the 675 survey responses received, 18 (2.3%) were identified as likely bots or fraudulent responses based on fraudulent screenshots or failed reCAPTCHA (reverse Completely Automated Public Turing Test to Tell Computers and Humans Apart), and 13 (1.6%) exhibited suspiciously high average session lengths (>3× the IQR, 70.35 minutes). An additional 22 participants (2.8%) timed out before completing the survey, 25 (3.1%) failed attention checks, 59 (7.4%) failed screenshot checks, 86 (10.75%) responded twice, and 21 (2.6%) declined to complete the survey. These categories were not mutually exclusive. The resulting sample consisted of 563 (70.4%) participants who consented and completed the full survey. Finally, 27 (4.8%) multivariate outliers were excluded according to the preregistration, yielding a final sample of 536 participants.

### Measures

#### Engagement

To verify the reliability of self-reported information, we collected both subjective self-reports and objective, app-verified data (screenshots provided by participants), which included minutes, days, streaks (consecutive days of use), number of sessions, average session length, and duration of app ownership (Table 1). These metrics were collected using recent app duration, defined as the number of days between first and last app use. App-verified duration was recorded if a participant validated the download period via screenshot.

The primary engagement variable was minutes of app use. Four variations were analyzed: (1) objective unadjusted minutes, representing total app-verified minutes; (2) self-reported or “subjective” unadjusted minutes, representing total unverified self-reported minutes; (3) objective adjusted minutes, calculated as app-verified minutes adjusted for app-verified duration of use, expressed as minutes per year; and (4) self-reported or “subjective” adjusted minutes, calculated as self-reported minutes adjusted for self-reported duration of use, expressed as minutes per year. Adjusted variables accounted for the duration of access to the app (from the download date to the last use). As only a limited number of apps reported objective start dates, and app-verified duration correlated highly with self-reported duration, the adjusted variables were calculated using the time between the first and last reported use.

**Table 1:** Descriptive statistics for engagement outcomes among meditation app users.

Statistics		n	Mean (SD)	5th percentile	25th percentile	50th percentile (median)	75th percentile	95th percentile
**Subjective**								
	Total minutes	483	3562.78 (8616.39)	4.10	76.00	420.00	2474.00	21735.10
	Total sessions	452	108.43 (197.00)	0.58	9.40	37.33	130.30	407.25
	Duration (days)	477	894.16 (854.20)	16.80	158.00	621.00	1342.00	2689.80
	Minutes per session	454	16.60 (29.74)	3.00	5.81	10.51	18.09	35.41
	Estimated minutes per month^a^	477	148.77 (304.56)	0.48	8.41	40.25	137.20	789.44
	Estimated sessions per month^a^	437	9.35 (16.61)	0.13	0.93	3.29	11.05	34.79
**Objective**								
	Total minutes	483	3358.69 (8607.36)	12.00	96.50	465.00	2410.00	21735.10
	Total sessions	151	61.89 (194.90)	0.61	3.36	12.22	39.95	167.23
	Minutes per session	151	49.21 (89.20)	1.98	7.26	25.92	55.90	154.08
	Estimated minutes per month^a^	151	73.58 (223.73)	0.61	2.39	10.61	35.09	313.31
	Estimated sessions per month^a^	148	9.44 (47.63)	0.02	0.10	0.66	2.32	17.29

^a^Estimated minutes per month and sessions per month were calculated by total engagement in minutes divided by duration of app use in years divided by 12.

#### Self-Reported Measures

##### Sociodemographic Information and Meditation History

Sociodemographic information included household income, education level, religion, app name, and approximate start and stop dates of use. Most data were self-reported via the survey. Prolific provided additional information, including age, sex, language, student and employment status, country of birth, and current residence. Regular practice information included minutes per session, sessions per day, and days per week. Meditation history was assessed by asking participants to report their previous meditation experience in hours, ranging from 0-100 hours to 1000+ hours. See Table 1 for regular practice information, and Sections S3-S5 in Multimedia Appendix 1 for sociodemographic statistics, meditation app frequencies, and a detailed survey flow.

##### Attention Checks

Three attention checks were included in the survey to assess participant engagement. Participants failing 2 or more attention checks were excluded. The attention checks were designed to mimic the scale items within which they appeared; for example, “In general, select dissatisfied to show that you are paying attention.”

##### The EuroQoL Health and Wellbeing Assessment—Short Form

The 9-item EuroQoL Health and Wellbeing (EQ-HWB-9) is a newly developed quality-of-life measure by Brazier and colleagues [57]. It assesses quality of life with a focus on health and well-being. The scale consists of 9 items, each rated from 1 (no difficulty, none of the time, and no physical pain) to 5 (unable, most or all of the time, and very severe physical pain). In this study, the 9 items of the EQ-HWB-9 demonstrated very good internal consistency (Cronbach α=0.873), and McDonald hierarchical omega was relatively high (ω=0.732). The EQ-HWB-9 was used with permission from the EuroQol Group.

##### The Kessler Psychological Distress Scale

The Kessler Psychological Distress Scale (K10) assesses psychological distress over the past 30 days [58]. This 10-item questionnaire, measuring anxiety and depressive symptoms, uses a 5-point scale ranging from 1 (none of the time) to 5 (all of the time). In this study, the scale demonstrated excellent internal consistency (Cronbach α=0.930; McDonald ω=0.799).

##### The Warwick-Edinburgh Mental Wellbeing Scale

The Short Warwick-Edinburgh Mental Wellbeing Scale (WEMWBS) was used to assess positive aspects of mental health [59]. This 7-item scale uses a 5-point response format, ranging from 1 (none of the time) to 5 (all of the time). In this study, the scale demonstrated high internal consistency (Cronbach α=0.879; McDonald ω=0.790).

##### The Satisfaction with Life Survey (Single Item)

The Satisfaction with Life Survey Single Item is an abbreviated version of the established Satisfaction with Life Survey (SWLS) [60]. The scale demonstrates reasonable criterion validity with the full SWLS (zero-order *r*=0.62-0.64). This single-item measure asks participants to rate their life satisfaction on a scale from 1 (extremely dissatisfied) to 7 (extremely satisfied).

##### 7-Item Generalized Anxiety Disorder Scale

The 7-Item Generalized Anxiety Disorder Scale is used to screen for general anxiety symptoms [61]. Each item is rated from 0 (not at all) to 3 (nearly every day). In this study, the scale demonstrated high internal consistency (Cronbach α=0.899; McDonald ω=0.863).

##### Depression (8-Item Patient Health Questionnaire)

The 8-item Patient Health Questionnaire is used to assess depressive symptoms [62]. Each item is rated from 0 (not at all) to 3 (nearly every day). In this study, the scale demonstrated high internal consistency (Cronbach α=0.881; McDonald ω=0.781).

##### Self-Compassion Scale

The Self-Compassion Scale consists of 12 items assessing participants’ ability to be compassionate toward themselves [63]. Each item is rated from 1 (almost never) to 5 (almost always). In this study, the scale demonstrated high internal consistency (Cronbach α=0.881; McDonald ω=0.656).

##### Self-Efficacy (6-Item Generalized Self-Efficacy)

The 6-item Generalized Self-Efficacy assesses self-efficacy, or an individual’s perceived ability to achieve goals [64]. Each item is rated from 1 (not at all true) to 3 (exactly true). The scale demonstrated high internal consistency (Cronbach α=0.821; McDonald ω=0.788).

##### Readiness to Change 1-Item

The Readiness to Change 1-item assessment is a 10-point scale that measures an individual’s preparedness to enact a behavioral change [37]. The scale ranges from 0 (not prepared to change) to 10 (already changing). It has been validated to reflect actual readiness in clinical contexts [37,65,66].

##### 6-Item Digital Working Alliance Inventory

The 6-item Digital Working Alliance Inventory (DWAI-6) [5] is a rating scale that assesses the therapeutic alliance between an individual and their health care provider, adapted for smartphone interventions (ie, referring to “the app” rather than “the therapist”). Items are rated on a 7-point scale from 1 (strongly disagree) to 7 (strongly agree), with subscales evaluating goal alliance (agreement on goals), task alliance (agreement on tasks), and bond (connection between app and user). The overall scale demonstrated good internal consistency (Cronbach α=0.850; McDonald ω=0.830). The Goal subscale showed high consistency (Cronbach α=0.766), the Bond subscale demonstrated acceptable consistency (Cronbach α=0.676), and the Task subscale showed poor consistency (Cronbach α=0.402).

##### Common Factors Domains (Modum Process Outcome Questionnaire)

We used a subset of items from the Common Therapeutic Relationship Factors Questionnaire (Modum Process Outcome Questionnaire) to assess the therapeutic relationship beyond the DWAI-6 [67]. The original questionnaire, which focuses on the clinician-patient relationship, was adapted to refer to the app-user relationship (eg, “I am able to be open and honest when interacting with the app”). Three items from the 12-item scale were included, each rated from 1 (strongly disagree) to 7 (strongly agree), with a “not applicable” option. The items demonstrated low internal consistency (Cronbach α=0.612; McDonald ω<0.001), likely due to being an unintended subset. Consequently, results will be reported for each item individually rather than as a total score.

##### The Big Five Inventory Short Form 2 (BFI-S-2)

The Big Five Inventory Short Form 2 (BFI-S-2) is a 30-item questionnaire assessing 5 personality domains: Extraversion, Agreeableness, Conscientiousness, Negative Emotionality/Neuroticism, and Open-Mindedness [68]. Each subscale consists of 6 items. Internal consistency, assessed using Cronbach α and McDonald ω, ranged from acceptable (Extraversion, α=0.766; ω=0.636/Agreeableness, α=0.744; ω=0.549/Openness, α=0.785; ω=0.664) to high (Conscientiousness, α=0.812; ω=0.738/Negative Emotionality, α=0.884; ω=0.841).

##### User Mobile Application Rating Scale

The user Mobile Application Rating Scale (uMARS) is a 27-item instrument for assessing the quality of mobile apps [69]. The scale includes subscales evaluating engagement (referred to as “appeal” in this study for clarity: “Is the app fun/entertaining to use?”), functionality (“How accurately and quickly do the app features and components work?”), aesthetics (“How good does the app look?”), information (“Is the app content correct, well written, and relevant to the goal/topic of the app?”), perceived/subjective quality (“Would you pay for this app?”), and perceived impact (“This app has increased my knowledge/understanding of meditating”). The scale asks participants to rate app elements on a 5-point scale ranging from 1 (poor) to 5 (excellent), with specific descriptions for each item. The overall scale demonstrated high internal consistency (Cronbach α=0.866) but only moderate reliability (McDonald ω=0.606). The additional subscale for perceived impact was also highly consistent (Cronbach α=0.863; McDonald ω=0.761). The Functionality subscale demonstrated high internal consistency and reliability (Cronbach α=0.803; McDonald ω=0.739), whereas the Engagement (Cronbach α=0.734; McDonald ω=0.628), Aesthetics (Cronbach α=0.761; McDonald ω=0.658), and Information (Cronbach α=0.762; McDonald ω=0.688) subscales showed acceptable consistency and reliability. The Subjective Quality subscale was consistent (Cronbach α=0.629; McDonald ω=0.667). The Aesthetics subscale showed variable internal consistency (Cronbach α=0.762; McDonald ω=0.065), indicating unequal item contributions.

### Analysis Plan

#### Sample Size Determination

Given an unknown effect size due to the absence of robust data, and guided by prior estimates of effect sizes for meditation apps [12], we aimed to calculate statistical power based on the smallest effect size we could reasonably detect. The target sample size was 1000 participants, providing 90% power to detect an effect of *r*=0.102, corresponding to a small effect. Because of recruitment challenges, the final sample size before analyzing the main engagement variable was 536, representing nearly 54% of our target population (n=1000). Although this reduction decreased statistical power (80% power to detect *r*=0.122), given that the a priori effect size was unknown, the study remained adequately powered to detect relatively small effects, albeit slightly larger than initially anticipated.

#### Planned Statistics

**Figure 1:**
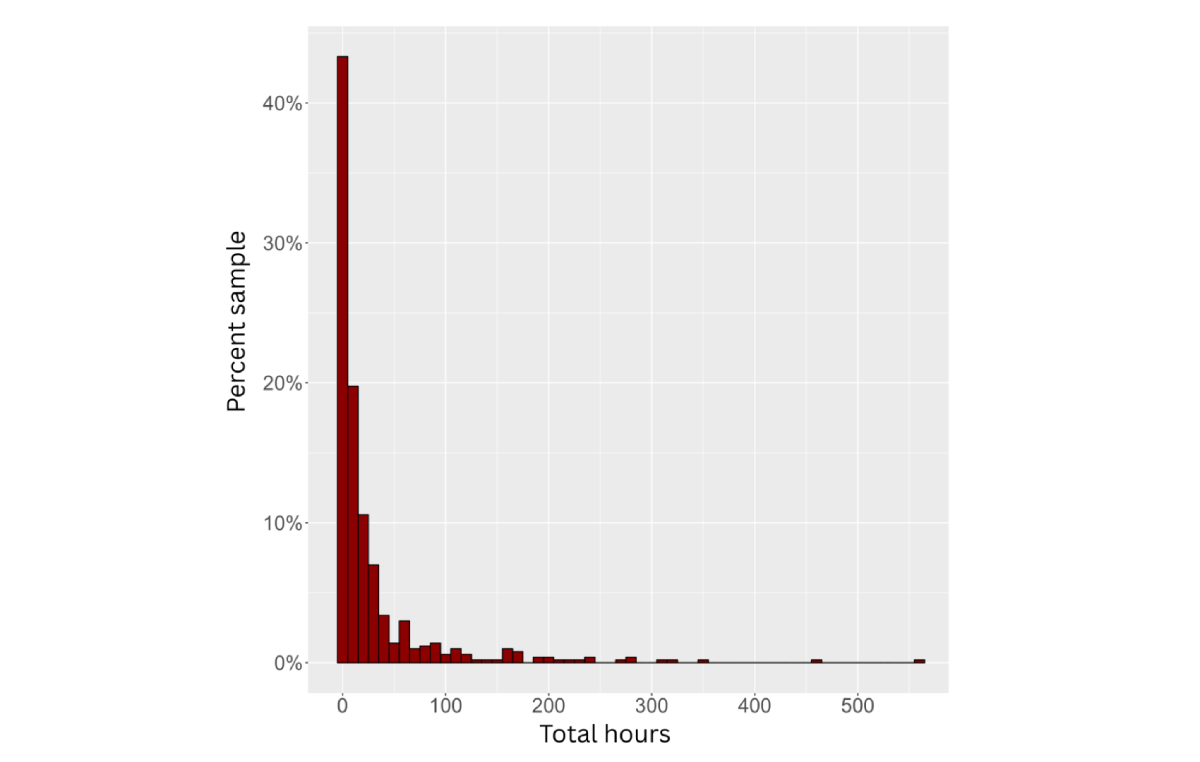
Histogram of self-reported practice hours (x axis) shown as the percentage of the sample (y axis), with values converted from minutes for visualization purposes.

As outlined in the preregistration, we explored associations between user-related factors (Q1, H1), user-app relationship factors (Q2, H2), app-related factors (Q3, H3), mental health factors (Q5, H5), and predefined engagement variables. All engagement variables were heavily skewed and nonnormal (see Figure 1 and Sections S11-S14 in Multimedia Appendix 1); therefore, Spearman rho correlations were estimated. Variables that were significantly associated with any outcome variable were subsequently used to build regression models for each of the 4 outcomes. As transformations did not normalize the variables, untransformed variables were analyzed using robust regression with the “robustbase” package in R (R Foundation), employing an MM-type regression estimator with a bisquare redescending score function [70,71]. This method applies case weighting to account for nonnormality. All analyses were conducted in RStudio (R Foundation).

#### Regression Models

Robust linear regression was used to investigate which factors accounted for significant variance in engagement. For each of the 4 outcome variables—adjusted objective minutes, adjusted self-report minutes, objective minutes, and self-report minutes—a separate regression model was created using the respective measure as the outcome variable. No stepwise regression was implemented. Instead, independent variables were selected from user, mental health, and app factors that were significantly associated with at least one outcome measure in the correlation analyses, following correction for multiple comparisons.

#### Multiple Comparisons Correction

We explored correlations between the 4 engagement outcomes and related factors, applying a false discovery rate (FDR) correction to account for multiple comparisons [72].

#### Data Cleaning

Duplicate and invalid responses were removed. The data demonstrated weak correlations, positive skew, and extreme values, indicating potentially high variability or inconsistency (see Sections S6-S10 in Multimedia Appendix 1). After adjusting for app duration, we implemented several data-cleaning procedures. Intraindividual response validity calculation, LongString Identification [73], and inconsistency of responses on the BFI-S-2 [68] were each used to identify and exclude extreme cases; however, none of these approaches resulted in major changes to the results (see Sections S10-S12 in Multimedia Appendix 1). As specified in the preregistration, multivariate outliers were removed, identified as cases with a Mahalanobis distance greater than the 95th percentile on the BFI-S-2 (see Section 13 in Multimedia Appendix 1).

## Results

### Overall Engagement

Overall, most users completed only a few minutes across limited sessions. Despite generally low engagement, 134 out of 536 (25%) users reported more than 11 sessions per month (approximately 1 session every 3 days), while the top 5% (27/536) reported around 35 sessions per month (more than 1 session per day). These patterns align with prior findings, including a median of 90% of users dropping off within the first week of real-world use and an average 42% drop in participation in meditation app randomized controlled trials spanning 1-2 months [4,12]. Notably, 402 (75%) participants reported more than 9 sessions, which contrasts with prior findings suggesting that most users disengage completely within a week of download. However, only the top 5% (25/536, 4.7%) engaged at levels consistent with clinically meaningful change [16], while the top 25% (134/536) engaged at levels comparable to the dose of mindfulness-based interventions [27].

### Participant Characteristics: Descriptives

#### Sociodemographic Features

**Figure 2:**
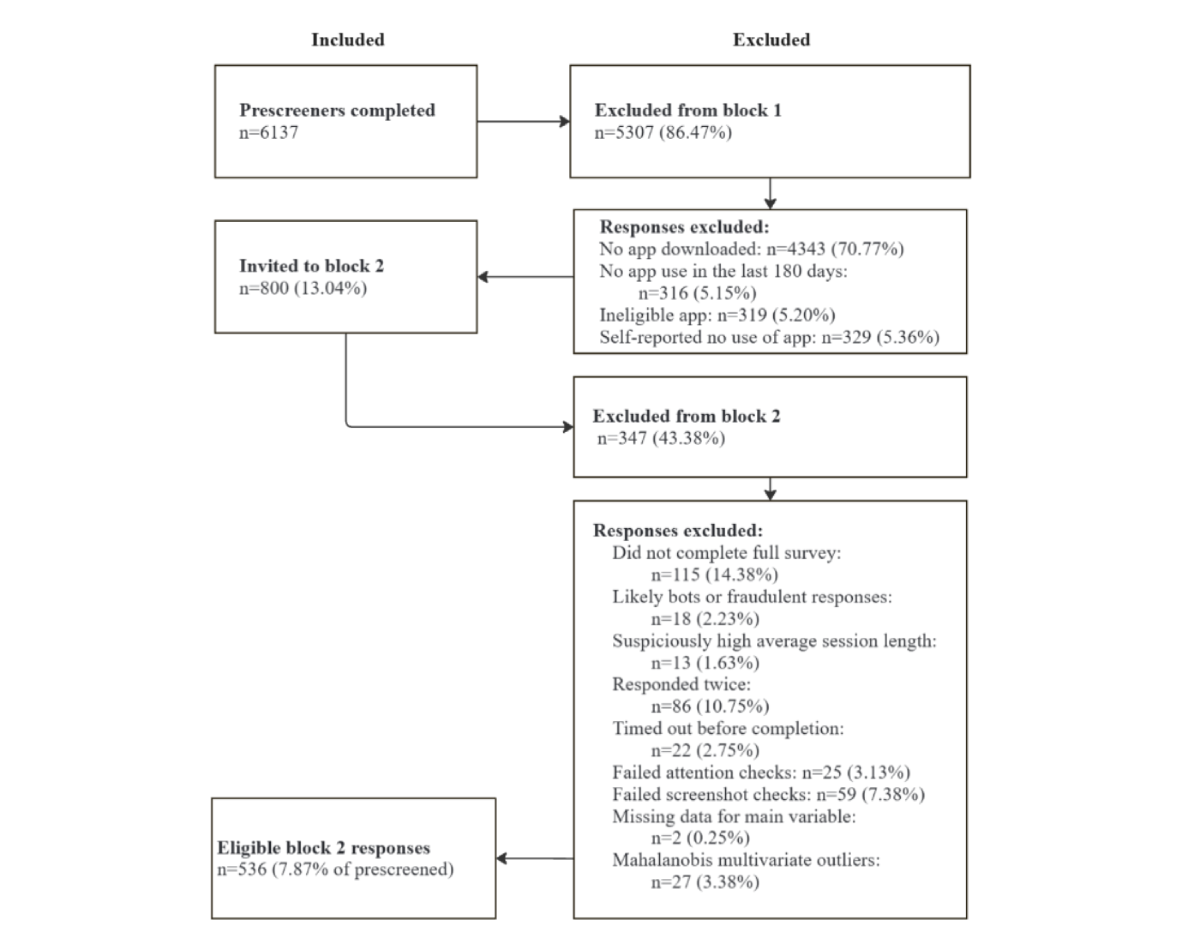
Participant flow.

Participants (N=536) ranged from 18 to 70 years of age (mean 36.56 years, SD 10.68 years) and were predominantly female (n=366, 68.3%). See Figure 2 for participant flow. Most resided in the United States 253 (47.20%) or the United Kingdom (n=226, 42.2%), with smaller proportions from Australia (n=27, 5%), Canada (n=21, 3.9%), and New Zealand (n=6, 1.1%). The majority identified as White (n=422, 78.73%) and were highly educated, with 387 (72.20%) holding at least a bachelor’s degree. Participants were also relatively wealthy, with nearly one-third reporting a combined income of $100,000 or more (n=145, 27.05%). Note that income brackets were not adjusted across countries. Nearly half of the participants reported no religious affiliation (n=264, 49.25%). The most frequently used apps were Headspace (n=191, 35.6%) and Calm (n=123, 22.9%), which together accounted for 58.6% of the sample (n=314). Full details are provided in Sections S3 and S4 in Multimedia Appendix 1.

#### Meditation Experience

Most users (n=330, 61.6%) reported between 0 and 100 hours of overall meditation experience. Meditation experience varied across meditation apps (*χ*^2^_4_=34.18, *P*<.001); users with 0-100 hours were most likely to use Headspace (124/377, 32.9%), followed by Calm (75/377, 19.9%) and Insight Timer (25/377, 6.6%; see Table 2).

**Table 2:** Frequency and relative percentage of Calm, Headspace, and Insight Timer users by self-reported meditation experience level (n=377).

Duration (hours)	Calm, n (%)	Headspace, n (%)	Insight Timer, n (%)	Total, n (%)
0-100	75 (19.9)	124 (32.9)	25 (6.6)	224 (59.4)
101-1000	35 (9.3)	46 (12.2)	46 (12.2)	127 (33.7)
1001+	6 (1.6)	11 (2.9)	9 (2.4)	26 (6.9)
Total	116 (30.8)	181 (48.0)	80 (21.2)	377 (100)

#### Engagement

**Figure 3:**
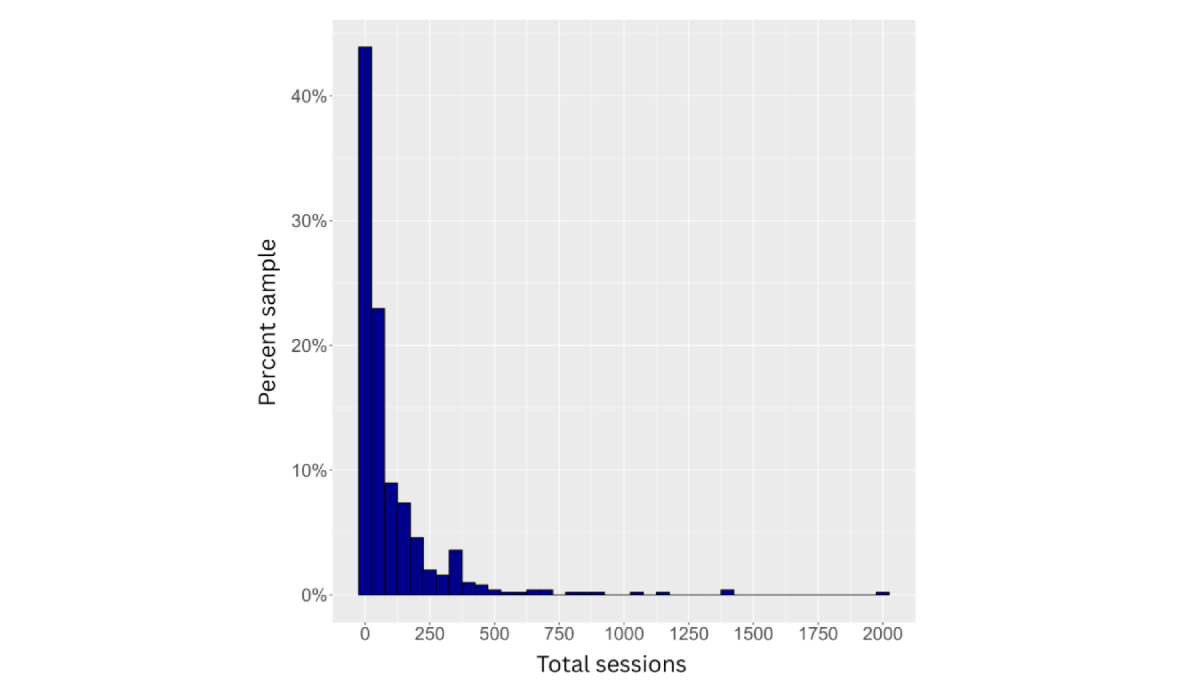
Histogram showing the total number of self-reported sessions (x axis) by the percentage of participants (y axis).

**Figure 4:**
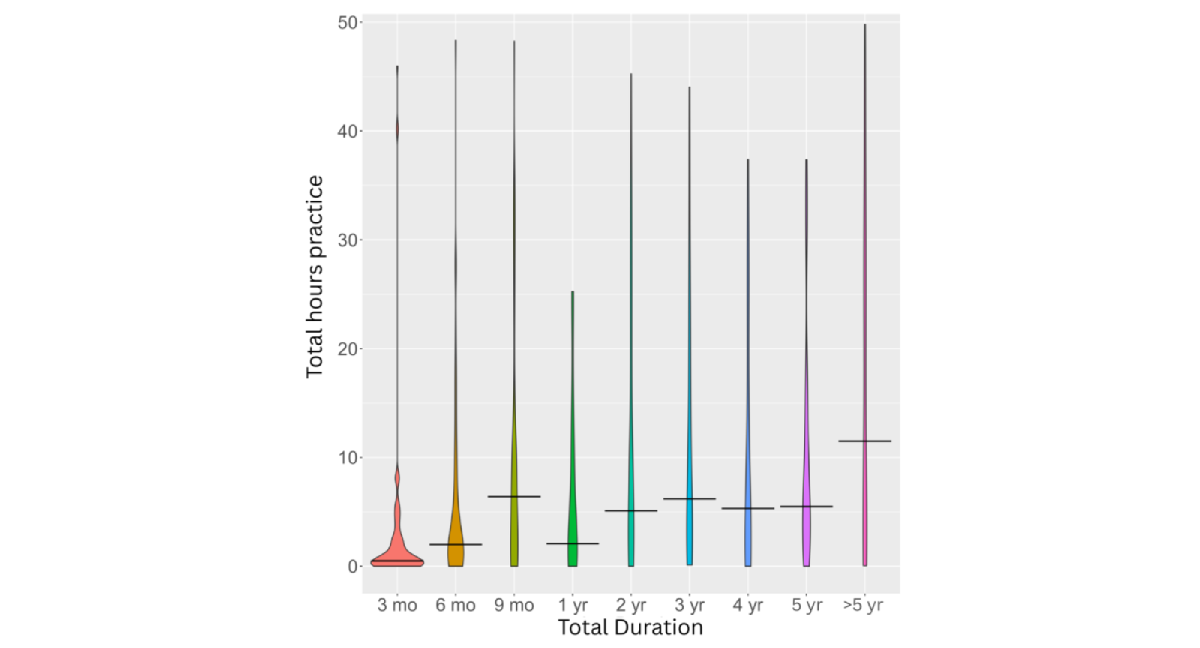
Total hours of practice by categorical groups based on time since app download. The y axis is truncated at 50 for visualization purposes, capturing 75% of the data. Horizontal bars indicate the median value for each category.

**Figure 5:**
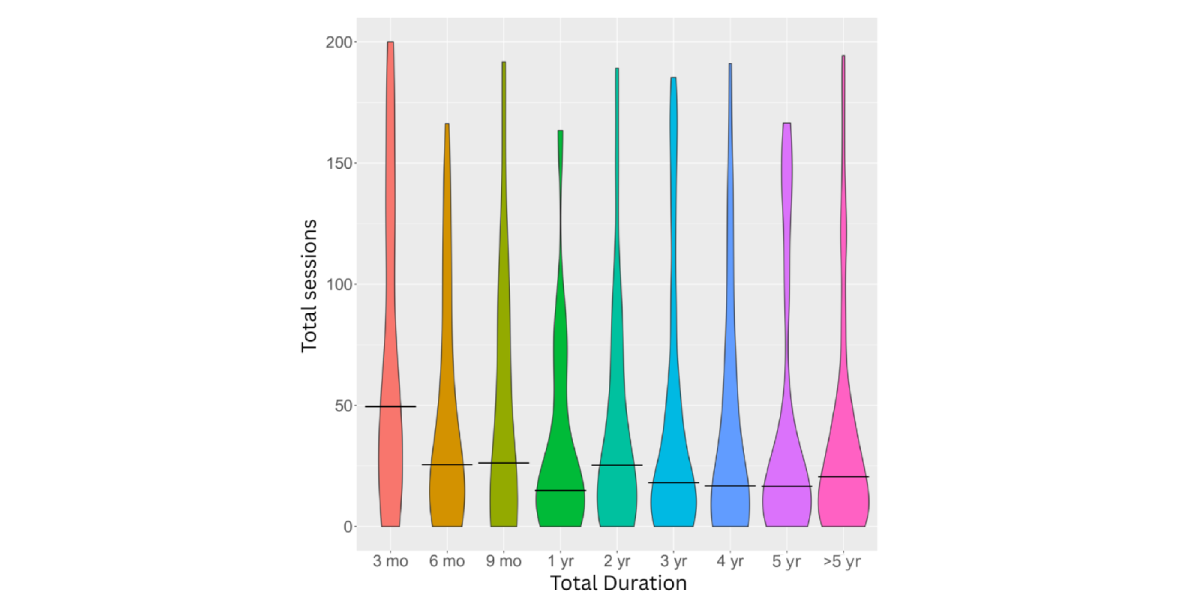
Total sessions by categorical groups based on time since app download. The y axis has been truncated for visualization purposes, capturing 75% of the data. Horizontal bars indicate median values for each category.

After excluding invalid responses and duplicates, engagement levels remained low, with a positive skew for both hours and sessions (see Figures 2 and 3). Adjusting for app duration showed low engagement regardless of how long the app had been available to users (see Figures 4 and 5). Estimated minutes per month and sessions per month were adjusted within each user to provide clearer engagement metrics. The median number of sessions per month was 3.29. With a median of 40.2 minutes per month, this equated to roughly three 12-minute sessions.

#### Engagement Statistics

Engagement variables were highly intercorrelated (*r*=0.495-0.999; see Section S14 in Multimedia Appendix 1). Objective adjusted minutes were the most reliable variable, but the sample was small and limited to 2 apps (n=156; Headspace and Waking Up). By contrast, subjective minutes with a subjective start date had a sample size 3 times larger (n=536). Given the strong association between objective and subjective start dates (*r*=0.783, *P*<.001), adjusted subjective minutes were calculated using subjective duration as the denominator to maximize sample size.

### Categorical Demographic Factors and Engagement

Categorical demographic associations with engagement are reported in Table 3. Being female was associated with lower engagement on 1 outcome, while residing in the United Kingdom was associated with higher engagement across 3 of the 4 outcome variables.

**Table 3:** Point-biserial correlations for the association between noncontinuous variables and engagement.

Variables			Objective minutes		Subjective minutes		Adjusted objective		Adjusted subjective
Sex^b^			–0.019		0.080		–0.296^a^		–0.016
**Residence**									
	Australia	–0.027		–0.016		–0.004		–0.040	
	United States	–0.071		–0.069		–0.065		–0.068	
	United Kingdom	0.102^a^		0.110^a^		0.075		0.106^b^	
	Canada	–0.032		–0.062		–0.029		–0.068	
	New Zealand	–0.021		–0.031		–0.007		0.038	

^a^*P*<.05 without multiple comparisons correction.

^b^*P*<.05 with multiple comparisons correction. For the biserial sex correlation, 1=female, 0=male.

### Engagement by App

Three 1-way analyses of variance indicated a significant effect of app type on engagement for subjective minutes (*F*_2,362_=9.03, *P*=.002, η^2^=.05) and adjusted subjective minutes (*F*_2,358_=7.63, *P*=.001; see Sections S15 and S16 in Multimedia Appendix 1). No significant differences were found for objective minutes (*F*_2,362_=0.972, *P*=.38).

### User Factors

**Figure 6:**
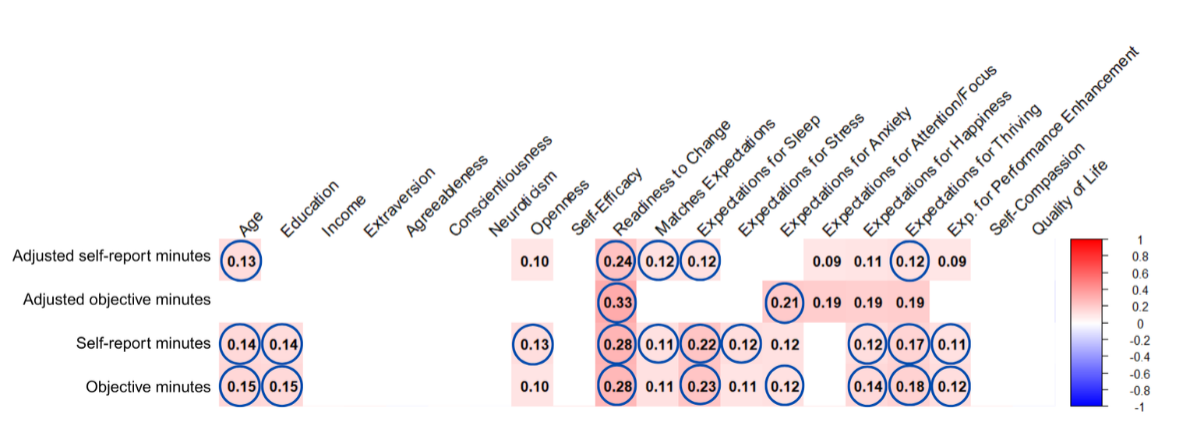
Heatmap of correlation results for user factors and adjusted self-report, adjusted objective, total self-report, and total objective minutes of meditation app use. The heatmap illustrates the direction and magnitude of Spearman correlations, as shown in the legend. Numbers indicate correlation coefficients. Asterisks denote correlations significant at *P*<.05 after correction for multiple comparisons. Nonsignificant correlations are omitted. Outcomes include self-reported total minutes of meditation app use, self-reported minutes adjusted for app duration, objectively verified total minutes of use (via app screenshot), and objective minutes adjusted for app duration.

We defined robust associations as those present across 3 or more of the 4 engagement outcomes. Among user factors, only 9 of 20 variables met this criterion (see Figure 6): age, openness (BFI-S-2), readiness to change, expectation match, expectations for sleep, expectations for anxiety, expectations for happiness, expectations for thriving, and expectations for performance enhancement. After FDR correction, only 4 of 20 remained: age, readiness to change, expectations for sleep, and expectations for thriving (see Section 17 in Multimedia Appendix 1 for CIs).

### Mental Health Factors

**Figure 7:**
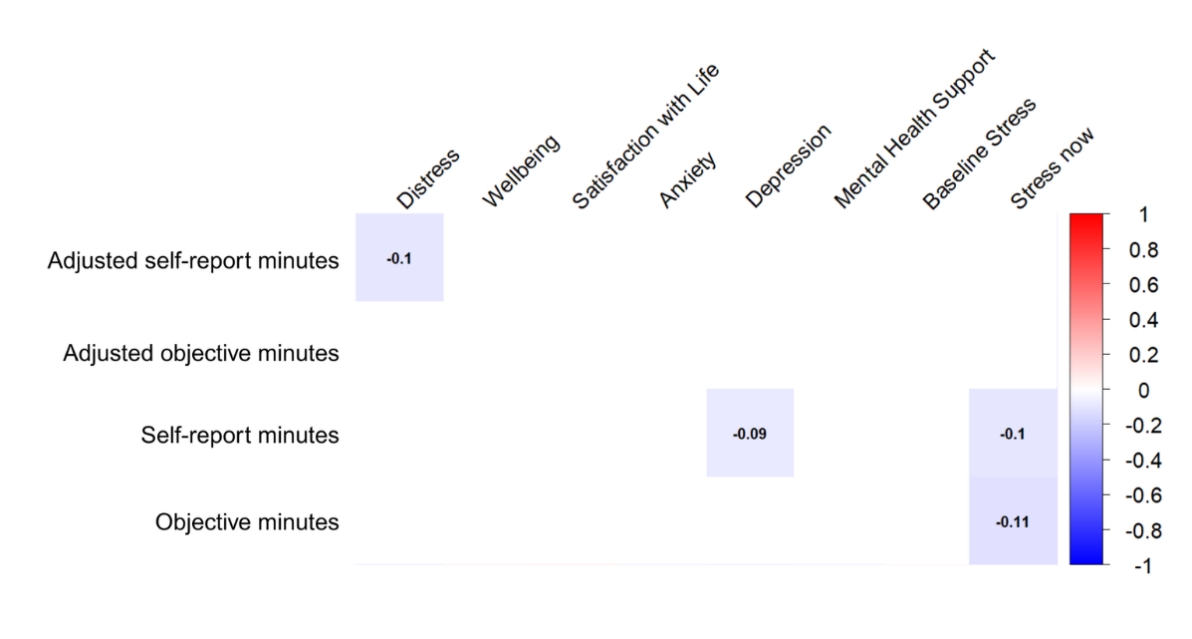
Heatmap of correlations between mental health factors and adjusted self-report, adjusted objective, total self-report, and total objective minutes of meditation app use. The heatmap shows the direction and magnitude of Spearman correlations, as indicated by the legend. Numbers represent correlation coefficients. Circles denote significant correlations at *P*<.05 after correction for multiple comparisons. Nonsignificant correlations are omitted. Outcomes include self-reported total minutes of meditation app use, self-reported minutes adjusted for app duration, objectively verified total minutes of use (via app screenshot), and objective minutes adjusted for app duration.

Self-reported stress, depression, and psychological distress were negatively associated with app use. Specifically, distress was negatively associated with adjusted self-reported minutes, depression with unadjusted self-reported minutes, and current stress with both unadjusted self-reported minutes and objective minutes. However, no mental health factors remained significantly associated with any engagement outcome after correction for multiple comparisons (see Figure 7).

### App Factors

**Figure 8:**
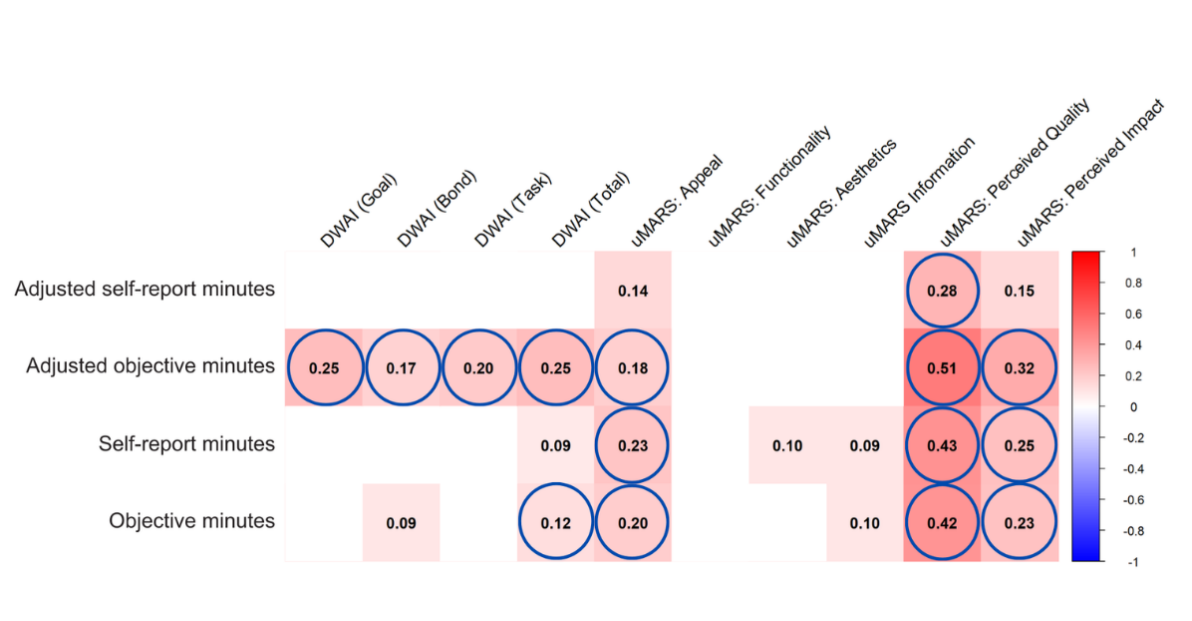
Heatmap of correlations between app factors and meditation app use outcomes. Displayed are Spearman correlation coefficients (numbers) showing the direction and magnitude of associations as indicated in the legend. Circles denote correlations significant at *P*<.05 after multiple comparison correction, and nonsignificant correlations are omitted. Outcomes include self-reported total minutes of app use, self-reported minutes adjusted for app duration, objective total minutes of use (verified via app screenshot), and objective minutes adjusted for app duration. DWAI: Digital Working Alliance Inventory; uMARS: user Mobile Application Rating Scale.

DWAI-6 Total, uMARS Appeal, uMARS Perceived Quality, and Perceived Impact were associated with 3 of the 4 outcome variables. Of the app factors investigated, 7 were associated with at least one engagement outcome after FDR correction (see Figure 8): DWAI-6 Total, as well as Goal, Bond, and Task subscales, and uMARS Appeal, Perceived Quality, and Perceived Impact.

### Outcome Regression Models

All models demonstrated a reasonable fit, explaining 12%-16% of the variance (see Table 4 and Section 18 in Multimedia Appendix 1). Significant (*P*<.05) predictors in 1 or more regression models included education, readiness to change, expectations for sleep, expectation match, the Perceived Quality subscale of the uMARS, and the Openness subscale of the BFI-S-2.

**Table 4:** Regression model coefficients for adjusted objective minutes, total objective minutes, adjusted self-report minutes, and total self-report minutes.

Standardized β coefficients for predictors in models 1–4			Adjusted objective minutes^a^, standardized β coefficients		Objective minutes^b^, standardized β coefficients		Adjusted self-report minutes^c^, standardized β coefficients		Subjective minutes^d^, standardized β coefficients
Intercept			–0.260^e^		–0.228^e^		–0.355^e^		–0.371^e^
**User factors**									
	Sex	0.001		<0.001		–0.008		0.002	
	Country of residence (United Kingdom)	<.0001		0.002		0.008		0.004	
	Age	0.003		<–.001		0.004		–0.001	
	Education	0.033		0.153^e^		0.532^e^		0.237^e^	
	Big Five Inventory Short form 2 openness	0.010^f^		0.003		0.005		0.006	
	Readiness to change	<0.001		0.005^f^		0.027^g^		0.008^f^	
	Expectations (match)	<0.001		–0.003		0.023^f^		–0.004	
	Expectations for sleep	–0.002		0.004		–0.008		0.009^f^	
	Expectations for stress	<0.001		<–.001		0.012		<.001	
	Expectations for anxiety	0.001		–0.001		–0.020		–0.003	
	Expectations for happiness	–0.005		0.002		0.009		<.001	
	Expectations for thriving	0.008		0.003		–0.006		0.006	
	Expectations for performance enhancement	–0.004		–0.003		–0.015		–0.003	
**App factors**									
	DWAI-6^h^ (Goal)	–0.009		0.004		–0.020		0.008	
	DWAI-6 (Bond)	–0.004		–0.003		–0.013		–0.002	
	DWAI-6 (Task)	0.010		0.002		0.024		0.004	
	uMARS^i^ (Appeal)	–0.012		<.001		–0.016		0.005	
	uMARS (Perceived Quality)	0.025^g^		0.003^g^		0.041^e^		0.010^f^	
	uMARS (Perceived Impact)	–0.006		<–.001		–0.003		0.002	
Adjusted *R*^2^			0.158		0.150		0.126		0.137

^a^App-verified minutes of use per year adjusted for total duration of use in years.

^b^Total app-verified minutes of use.

^c^Self-report minutes of use per year adjusted for total duration of use in years.

^d^Self-report total minutes of use.

^e^*P*<.001.

^f^*P*<.05.

^g^*P*<.01.

^h^DWAI-6: 6-item Digital Working Alliance Inventory.

^i^uMARS: user Mobile Application Rating Scale.

## Discussion

### Principal Findings

We examined factors associated with engagement in popular meditation apps among 536 participants. Consistent with prior findings, most participants engaged minimally. Although apps were available for an average of 894 days (about 2.5 years), participants reported an average of 108 sessions, while app-verified data from about one-third of participants indicated 62 sessions on average. Half of the sample engaged in 3 or fewer sessions per month. Notably, engagement did not increase with longer app availability, suggesting a pattern of persistently low overall engagement.

Few significant correlations between individual user factors and engagement were observed, most of which were small in magnitude (*r*=0.09-0.30), with a few reaching the moderate range (*r*=0.30-0.50). After correction for multiple comparisons, positive associations with engagement remained for male sex, older age, higher education level, readiness to change, and expectations of the app for stress reduction, sleep improvement, anxiety reduction, happiness, thriving, and performance enhancement. These results suggest that older, more educated users with greater readiness to change and higher expectations of the app are more likely to engage regularly. App factors—including perceived appeal, quality, and impact—were consistently associated with higher engagement, as were the 3 digital working alliance subscales (Goal, Task, and Bond). This indicates that both perceptions of the app and the perceived relationship with it may be important determinants of engagement.

### User Factors Related to Use

#### Sociodemographics

Education was the variable most consistently associated with higher engagement. Meditation is more common among individuals who are White, middle-aged, wealthier, and better educated [41]. Lower levels of education have been linked to earlier disengagement or a failure to engage in meditation at all [41]. Higher education is also associated with greater engagement in mindfulness practices in US nationally representative surveys [11]. Lower levels of education have also been linked to poorer health outcomes [74,75] and are related to health literacy, which partially mediates health-promoting behaviors [76,77]. While lower education may contribute to poorer health outcomes and lower health literacy, other social factors may also reduce engagement. For example, individuals with lower education often have more fragmented leisure time, leaving less opportunity for regular, recurrent activities [78]. Male sex was associated with 1 engagement measure, which contrasts with prior research showing that females are generally more likely to engage in meditation practice, even after controlling for other demographic factors [12,42,79]. Previous studies have also found that males may demonstrate greater persistence in meditation [80]. As this correlation was observed only for adjusted objective use (available for Headspace and Waking Up), it may reflect patterns specific to users of these apps rather than meditation app use more broadly [40]. Notably, sex did not emerge as a significant predictor in the regression models.

#### Personality

Of the personality factors, only openness was related to engagement. Openness reflects general curiosity and a willingness to explore novel perspectives of one’s subjective experience [81]. Individuals higher in openness are more likely to try meditation initially and persist despite encountering difficulties. Openness has also been associated with meditation practice outside of group meditation class settings [45]. In contrast to prior research, we found no associations between engagement and conscientiousness, extraversion, agreeableness, or neuroticism [55,82,83]. While conscientiousness was not related to engagement in this study, it has previously been linked to positive attitudes toward practice [43]. Similarly, neuroticism showed no association with engagement here, although prior work has linked it to perceiving more barriers to practice [84,85].

#### Mental Health

None of the 8 mental health factors were significantly associated with engagement. Previous research has found meditation apps to be modestly effective for depression and anxiety [12,19], potentially serving as a form of self-managed treatment for individuals facing barriers to mental health care [41]. However, no such associations with mental health factors were observed in this study. In a previous study, motivation for mental health was negatively associated with app use [40]. Meditation can negatively impact mental health [86]. While these meditation-related adverse events do not always result in impairment, about half of meditators report experiencing an adverse effect, and 9.1% report functional impairment as a result [86]. Individuals who do not experience benefits or who encounter adverse effects may disengage shortly after download. Furthermore, meditating for mental health reasons has been negatively associated with the total amount of meditation practice completed over the long term [27,87]. Individuals with higher lifetime meditation practice often shift toward spiritual motivations as their practice progresses [29]. However, the retrospective design of our study limits causal inferences.

### App Factors

#### uMARS

Five of the 6 uMARS subscales were associated with engagement. Previous research suggests that aesthetics and appeal relate to meditation app engagement [53], although in our study, aesthetics were not robustly associated after FDR correction. The Perceived Quality subscale showed the strongest association (*r*=0.51), indicating that user perceptions may drive both usage and beliefs in the app’s effectiveness. Perceived impact was also robustly associated with engagement. Given the retrospective design, survivorship bias should be considered: users who continued using the apps likely enjoyed them, while those who did not may have stopped. It is also possible that users who experienced benefits from their chosen app developed increasingly positive app appraisals over time.

#### Digital Working Alliance

The DWAI-6 Goal, Bond, and Task subscales, as well as the overall score, were associated with engagement, consistent with prior findings [88]. All subscales correlated with adjusted objective minutes—the most reliable outcome measure, computed using app-verified minutes and download date—but this could only be calculated for apps that provide download dates (Headspace and Waking Up; n=151). Therapeutic alliance and engagement may promote each other [88]. While therapeutic alliance is considered important in digital mental health [89], current measures are adaptations of traditional, human-centered alliance scales. Incorporating human-computer interaction perspectives may provide greater nuance, particularly for anthropomorphic scale items [90]. Despite this limitation, therapeutic alliance with apps remains relevant to engagement, as alignment between a user’s goals and perceived app support may encourage continued use.

One consideration for both the uMARS and DWAI-6 is that several subscales demonstrated relatively poor internal consistency. The reliability of the uMARS Engagement and Subjective Quality subscales, as well as the DWAI-6 Bond and Task subscales, ranged from acceptable to poor, which reduces confidence in the constructs being measured.

#### Expectations for Efficacy

Higher expectations of efficacy across 6 of the 7 domains assessed (sleep, stress, anxiety, happiness, thriving, and performance enhancement) were generally associated with higher engagement, with the exception of expectations for attention/focus. Only expectations for sleep were significant in the regression model. These findings align with our predictions. Experimental and prospective studies have shown that failing to meet expectations is more predictive of behavior than matched expectations [91,92]. Unmet or low expectations negatively influence engagement and perceived usefulness, whereas met or exceeded expectations positively affect behavior and perceptions [92]. Expectations are closely linked to app ratings, as features such as goal setting and feedback enhance beliefs in an app’s effectiveness [34]. These features can also foster positive experiences of progress, creating a feedback loop that promotes further engagement [33,48]. In the absence of human interaction, the relationship between a user and an app is shaped by the “user journey”—the path a user follows through the app’s design. Persuasive design can help establish and meet user expectations.

A general rating of whether expectations were met was weakly associated with engagement. This result aligns with literature suggesting that matched expectations have a positive influence on behavior and mismatched expectations have a negative effect [93]. It is worth noting that we asked, “To what extent did your experience match your initial expectations?” without specifying which expectations participants should consider. Consequently, this approach may have captured only an overall impression of expectation match.

#### Readiness to Change

Readiness to change showed robust, moderate associations and accounted for a significant proportion of variance in the regression model. The readiness-to-change ruler used in this study is actively employed in behavior change interventions and is based on the Transtheoretical Model of Change, which conceptualizes behavior change in stages [32,94,95]. Readiness to change shows promise as one of the most predictive factors of actual behavior change, as it is conceptually closely linked to both motivation and behavior. These findings align with broader evidence connecting readiness ratings to actual behavior change, particularly in health-related contexts [63,95,96]. This relationship could inform app design, allowing offerings to be tailored to users’ readiness levels. The same single-item measure used in our study could be implemented immediately after app download to tailor the length, complexity, and type of practice to users’ readiness levels. For example, users with lower readiness could be offered shorter, simpler meditations or psychoeducational content about meditation to reduce perceived barriers and enhance understanding of the practice.

### Self-Efficacy and App Ratings in Building Habits

Contrary to our expectations, self-efficacy was not related to engagement. Previous research on habit formation suggests that self-efficacy may support the maintenance of a target behavior before a habit is established. There is limited evidence that self-efficacy promotes habit-building [33,48,97] and increases with ongoing meditation practice [98]; however, results are mixed [99,100]. One likely reason self-efficacy did not predict engagement is that expectations, perceptions, and habit formation played larger roles. A person may believe they can achieve a goal, but if they are not committed or do not perceive long-term utility, they may lack motivation to engage. This may explain why readiness to change was associated with engagement, whereas self-efficacy was not.

### Limitations

One key limitation of this study is that its retrospective design precludes causal inferences, although research on meditation app engagement is generally scarce. Additionally, we cannot confirm detailed usage patterns, such as extended gaps or cessation points; however, our estimates of sessions per month provide a rough indication of practice regularity. This study included cross-app comparisons, which few prior studies have conducted. Such comparisons are valuable, given that all therapeutic alliance subscales and half of the uMARS subscales were associated with engagement after correction for multiple comparisons. However, by not focusing on a specific app, the sample was disproportionately composed of users of the most popular apps.

Another limitation was that our most reliable outcome variable—objective minutes adjusted for verified app duration—was restricted to apps that displayed the month or year of joining. As a result, the sample for objective-adjusted minutes comprised only about one-third of the self-reported sample. Nevertheless, objective minutes were highly correlated with self-reported minutes, which may mitigate some concerns, although it is possible that individuals who can view their app-recorded minutes rely on these records when self-reporting.

Our data quality may have been influenced by self-selection, socioeconomic skew, and the compensation structure in our Prolific sample. Nevertheless, research indicates that among popular online survey platforms, Prolific consistently provides high-quality data across a wide range of measures [101]. Additionally, our data may have been skewed by the overrepresentation of the most popular meditation apps, limiting the generalizability of the findings to less popular apps or those with a narrower focus.

A final significant issue concerns what the outcome measures captured. While meditation was the central focus of the included apps, many also offer alternative exercises that contribute to the measured minutes, including—but not limited to—breathwork, sleep stories, and podcasts. This is an issue because sleep stories may continue running for hours after an individual falls asleep and be recorded as meditation. Future studies that can distinguish between different activities will provide more accurate statistics on meditation engagement.

### Future Directions

A group-level comparison of engagers and disengagers could reveal cluster effects, where active users share similar characteristics. Baumel et al [4] observed a drop-off trend in a large sample but noted that understanding precisely why people engage or disengage during this period would be of interest. While there is a high use rate among those who continue engaging beyond the first week, this represents only a small portion of users. By including all users over the past 180 days, we obtain a general picture of app use across the population; however, this approach results in a large sample of disengagers and only a small sample of active users, limiting our power to detect effect sizes within the subsample of engagers.

Longitudinal analysis could directly examine temporal and causal aspects of engagement, account for changes in contributing factors over time, and provide a clearer understanding of baseline predictors. For example, longitudinal data could track whether changes in mental health outcomes influence engagement. This study did not find any significant associations for mental health outcomes that survived multiple comparisons. However, we relied on participants’ reports of mental health status following meditation app use. Apps have been shown to reduce outcomes such as stress, depression, and anxiety [12], and such changes could positively or negatively reinforce app use. Moods, circumstances, and lifestyles can fluctuate widely over extended periods. User ratings of apps using scales such as the uMARS may better explain engagement when app rating and user engagement occur close together. Longitudinal analysis also allows for baseline measurement of variables, such as expectations, which can then be compared with actual experiences at follow-up. The low proportion of variability accounted for suggests that factors outside the model have a significant impact on engagement.

### Conclusions

This study aimed to explore factors influencing engagement with popular meditation apps, highlighting a substantial early drop-off. Although the models accounted for only a small proportion of overall variance, the findings emphasize the importance of user characteristics and app quality in sustaining engagement. This exploratory study aimed to examine a wide range of factors potentially relevant to meditation app engagement. The results indicated that older, more educated users, as well as those with higher expectations of apps and greater readiness to change, were more likely to engage with the apps regularly.

## Appendix

Multimedia Appendix 1 Additional analysis.

## Abbreviations

BFI-S-2: Big Five Inventory Short Form 2
DWAI-6: 6-item Digital Working Alliance Inventory
EQ-HWB-9: 9-item EuroQoL Health and Wellbeing
FDR: false discovery rate
K10: Kessler Psychological Distress Scale
MBP: mindfulness-based program
reCAPTCHA: reverse Completely Automated Public Turing Test to Tell Computers and Humans Apart
SWLS: Satisfaction with Life Survey
uMARS: user Mobile Application Rating Scale
WEMWBS: Warwick-Edinburgh Mental Wellbeing Scale

## Data Availability

Data and analytic code are uploaded to the Open Science Framework registry [102].
