# Supplementary material for: Engagement With Meditation Apps: Cross-Sectional Survey of Use and Associations
Source: J Med Internet Res. 2026 Feb 2;28:e71960. doi: 10.2196/71960 (PMC12910276; doi:10.2196/71960)
Supplement: Multimedia Appendix 1 [file jmir_v28i1e71960_app1.docx]

**Supplementary Materials**

**Supplementary Methods**

**S1.**

**Informed Consent**

**Consent Form Melbourne School of Psychological Sciences**

Ethics ID Number: 23969

**Project**: Meditation Experiences Survey

**Responsible Researcher:** A/Prof Nicholas Van Dam

Tel: +61 3 8344 3644 Email: [nicholas.vandam@unimelb.edu.au](mailto:nicholas.vandam@unimelb.edu.au)

**Additional Researchers:**

Dr Julieta Galante (researcher) Email: [julieta.galante@unimelb.edu.au](mailto:julieta.galante@unimelb.edu.au)

Dr Jonathan Davies (researcher) Email: [jonathan.davies@unimelb.edu.au](mailto:jonathan.davies@unimelb.edu.au)

Dr Simon D'Alfonso (researcher) Email: [dalfonso@unimelb.edu.au](mailto:dalfonso@unimelb.edu.au)

Ms Felicity Miller (student researcher) Email: [familler@student.unimelb.edu.au](mailto:familler@student.unimelb.edu.au)

Ms Julia Adams (student researcher) Email: [juliaa1@student.unimelb.edu.au](mailto:juliaa1@student.unimelb.edu.au)

Ms Fangziyun Tong (student researcher): Email: [fangziyunt@student.unimelb.edu.au](mailto:fangziyunt@student.unimelb.edu.au)

Ms Yana Ryakhovskaya (student researcher) Email: [yryakhovskay@student.unimelb.edu.au](mailto:yryakhovskay@student.unimelb.edu.au)

Ms Jessica Targett (student researcher) Email: [jtargett@student.unimelb.edu.au](mailto:jtargett@student.unimelb.edu.au)

Mr Alex Burger (student researcher) Email: [alex.burger@unimelb.edu.au](mailto:alex.burger@unimelb.edu.au)

1. I consent to participate in this project, the details of which have been explained to me, and I have been provided with a written plain language statement to keep.
2. I understand that the purpose of this research is to understand what personal and app factors are associated with user experiences and perceptions of mindfulness apps.
3. I understand that my participation in this project is for research purposes only.
4. I acknowledge that the possible effects of participating in this research project have been explained to my satisfaction.
5. In this project I will be asked to answer a series of questions about who I am, my health and wellbeing, my meditation practice, my interest and engagement with mindfulness apps, and my experiences and perceptions of mindfulness apps as part of a 20-30 minute survey.
6. I understand that I will be paid US$0.20-0.40 for completing the screener survey (Part 1) and US$4-6 for completing the follow up survey (Part 2).
7. I understand that my participation is voluntary and that I am free to withdraw from this project anytime without explanation or prejudice and to withdraw any unprocessed data that I have provided.
8. I understand that the raw data from this research will be stored at the University of Melbourne. At the conclusion of the study, we will make the deidentified, cleaned and summarised data set publicly available in perpetuity via the Open Science Framework for use in future projects that are closely related to this project, the same general area, or could make valuable use of this data.
9. I have been informed that the confidentiality of the information I provide will be safeguarded subject to any legal requirements; my data will be password protected and accessible only by the named researchers.
10. I understand that after I sign and return this consent form, it will be retained by the researcher.

| **By clicking the tick box, I agree to participate** |  | **Date** |  |
| --- | --- | --- | --- |

**S2.**

**List of apps**

S2. List of apps included in the study.

Smiling Mind

Headspace

Insight Timer

Calm

Waking up

Medito

Ten Percent Happier

UCLA Mindful

Healthy Minds Program

Plum Village

Simple Habit

Petit Bambou

[Open text available for other apps]

**S3.**

**Demographics**

Table S1. Demographics (Detailed Table).

| Category | Subcategory (where applicable) | *Mean ± SD* |
| --- | --- | --- |
| Age |  | 36.46 **±** 10.68 |
| Meditation Experience (minutes) |  | 3565 **±** 8632.26 |
|  |  | *n (%)* |
| Sex | Male | 166 (30.97%) |
|  | Female | 366 (68.28%) |
|  | Prefer not to say | 1 (0.002%) |
|  | Missing | 1 (0.002%) |
| Ethnicity | White | 422 (78.73%) |
|  | Asian | 43 (8.022%) |
|  | Black | 26 (4.85%) |
|  | Mixed | 28 (5.22%) |
|  | Other | 15 (2.80%) |
|  | Missing | 3 (0.006%) |
| Education | Less than high school | 2 (0..002%) |
|  | High school (or equivalent) | 31 (5.78%) |
|  | Some college, no degree | 85 (15.86%) |
|  | Associate’s degree | 31 (5.78%%) |
|  | Bachelor's degree | 230 (42.91%) |
|  | Master's Degree | 123 (22.95%) |
|  | Professional School or Doctorate Degree (MD, PhD, JD, etc.) | 34 (6.34%) |
| Family Income | $0 - $34,999 | 94 (17.54%) |
|  | $35,000 - $49,999 | 78 (14.55%) |
|  | $50,000 - $74,999 | 106 (23.51) |
|  | $75,000 - $99,999 | 100 (18.66%) |
|  | $100,000 and over | 145 (27.05%) |
| Country of Residence | United States | 253 (47.20%) |
|  | United Kingdom | 226 (42.16%) |
|  | Australia | 27 (5%) |
|  | Canada | 21 (3.91%) |
|  | New Zealand | 6 (1.11%) |
| Meditation Experience | 0-100 hours | 330 (61.57%) |
|  | 100-1000 hours | 176 (14.18%) |
|  | 1001+ hours | 30 (45.60%) |

**Note**: Meditation experience was a self-reported estimate.

**S4**

Table **S2**. Top Meditation Apps Used.

| App | Number of Users (N(%)) (n = 536) |
| --- | --- |
| Headspace | 191 (35.63%) |
| Calm | 123 (22.95%) |
| Insight Timer | 82 (15.29%) |
| Balance | 38 (7.09%) |
| Healthy Minds | 27 (5.04%) |
| Medito | 20 (3.73%) |
| Waking Up | 21 (3.91%) |
| Apple Mindfulness | 12 (2.24%) |
| Smiling Mind | 7 (1.31%) |
| Peloton | 7 (1.31%) |
| Fitbit | 5 (0.09%) |
| Other | 3 (0.06%) |

**S5.**

**Detailed description of survey flow**

In the first block, information gathered included (1) past and present app use, (2) expectations for meditation apps' effectiveness for sleep, stress, anxiety, attention, happiness, thriving and performance enhancement, (3) most recent app used, (4) date of first and last app use, (5) usage statistics provided by the app. Participants were asked to upload a screenshot to verify reported use. Prolific IDs were gathered for payment purposes.

In the second block, in addition to completion of the scales cited below, participants provided the following - demographic information including (1) education level, (2) income level and (3) religious affiliation; general information gathered relating to meditation practice included (4) earliest meditation experience, (5) earliest meditation app experience, (6) total lifetime hours of meditation practice, (7) main general practice type (app, hybrid or non-app), (8) preference of practice space (alone or with others), (9) percentage of various styles of meditation,(10) percent of time using meditation apps regularly, (11, 12) practice frequency (days per week, times per day), and (13) practice duration in minutes.

In terms of meditation app motivations, participants were asked about their reasons for commencing and (if applicable) ceasing app use (14). Participants rated whether their use was or is (14.1) to address a specific problem, (14.2) to address an area where they could be better, (14.3) for general wellness and (14.4) to attain a greater goal on a scale of 1 (not at all) to 5 (very strong). Participants were also asked; (17) what external sources may have contributed to their use (e.g. recommended by a friend, advertisements), (18) why they stopped practicing (if applicable). Participants were given the option to provide free-text reports of reasons for discontinuing use or could select from common reasons reported in the literature. Next, participants were asked (19) whether any unpleasant experiences had occurred during meditation practice. If a participant indicated that they had experienced an unpleasant experience, they were asked (19.1) how intense it was, (19.2) how unpleasant it was, (19.3) whether the experience was the result of using an app, (19.4) when it happened, (19.5) whether function was impaired, (19.6) how long impairment lasted, (19.7) how practice may have changed as a result, and (19.8) how related it was to meditation practice.

General mental health information included (20) whether the participant had ever received professional mental health support, (20.1) when they received that support, (20.2) mental health state at app onset from 1 (healthy or well) to 4 (seriously or severely distressed) and stress levels at (20.3) app onset and (20.4) in the past week or two, on a scale from 1 (not at all) to 5 (very much).

Finally, information about health economics was gathered, assessing (21.1) whether any money was spent on meditation resources in the last 3 months, (21.2) how much was spent in USD, (21.3) in what ways a meditation practice has been perceived to save the participant for expenditures (e.g. on medical visits, therapy sessions), and (21.4) how much has perceived to have been saved as a function of either health-related visits or percent of reduced savings.

**S6.**

Normality Tests for Objective Minutes (unadjusted)

*Linearity Test*

A RESET test indicated a significant deviation from linearity, F (2,450) = 6.19, *p* = .002, suggesting that the model may benefit from non-linear terms.

*Normality Test*

The Shapiro-Wilk test showed non-normal distribution of residuals, *W* = 0.103, *p* < .001.

*Homoscedasticity Test*

The Breusch-Pagan test revealed homoscedasticity, *X^2^* = 29, df(14) = 14, *p* = 0.941.

*Multicollinearity Test*

Variance inflation factors (VIFs) were overall above the threshold, indicating multicollinearity issues.

*Highest VIF:*

DWAI (goal) = 7.622

These results suggest potential non-linearity, non-normal residuals, homoscedasticity, and significant multicollinearity.

**S7.**

Normality Tests for Objective Minutes (adjusted)

*Linearity Test*

The Ramsey Reset test indicated non-linearity or specification errors in the model, F (2, 123) = 31.32, *p* < .001, *p* < .001.

A RESET test indicated a significant deviation from linearity, F (2, 123) = 31.32, p < .001, suggesting that the model may benefit from incorporating non-linear terms.

*Normality Test*

The Shapiro-Wilk test showed non-normal distribution of residuals, *W* = 0.261, *p* < .001.

*Homoscedasticity Test*

The Breusch-Pagan test revealed homoscedasticity, *X^2^* = 18.977, df(19) = 14, *p* = .46.

*Multicollinearity Test*

Variance inflation factors (VIFs) was above the threshold (<5), indicating multicollinearity issues.

*Highest VIF:*

DWAI (task) = 19.24

These results suggest potential non-linearity, non-normal residuals, homoscedasticity, and significant multicollinearity.

**S8.**

Normality Tests for Subjective Minutes (unadjusted)

*Linearity Test*

A RESET (Regression Specification Error Test) was conducted to assess the specification of the model. The results indicated specification error, F (2,477) = 57.053, p <. 001.

### *Normality Test*

The Shapiro-Wilk test showed non-normal distribution of residuals, W = 0.44, *p* < .001.

### *Homoscedasticity Test*

The Breusch-Pagan test revealed heteroscedasticity, X^2^ = 45.5, *df(*17), *p* < .001.

### *Multicollinearity Test*

Variance inflation factors (VIFs) were all below the threshold of concern (< 5), indicating no severe multicollinearity issues.

*Highest VIF:*
DWAI (goal) = 8.68

These results suggest potential non-linearity, non-normal residuals, heteroscedasticity, and significant multicollinearity.

**S9.**

Normality Tests for Subjective minutes (adjusted)

### *Linearity Test*

A Ramsey RESET (Regression Specification Error Test) was conducted to evaluate the specification of the model. The results indicated a significant specification error, F(2,472) = 47.054, *p* < .001. This suggests that the model may be misspecified, indicating potential issues such as omitted variables or incorrect functional form.

### *Normality Test*

The Shapiro-Wilk test showed non-normal distribution of residuals, *W=* 0.496, *p* < .001.

*Homoscedasticity Test*

The Breusch-Pagan test revealed homoscedasticity, X^2^ = 52.386*,* df(19), *p* = 0.458.

### *Multicollinearity Test*

Variance inflation factors (VIFs) exceed the threshold (<5) indicated potential multicollinearity issues.

*Highest VIF:*

DWAI (task) = 6.981

**S10.**

Range of Intraindividual Response Variability


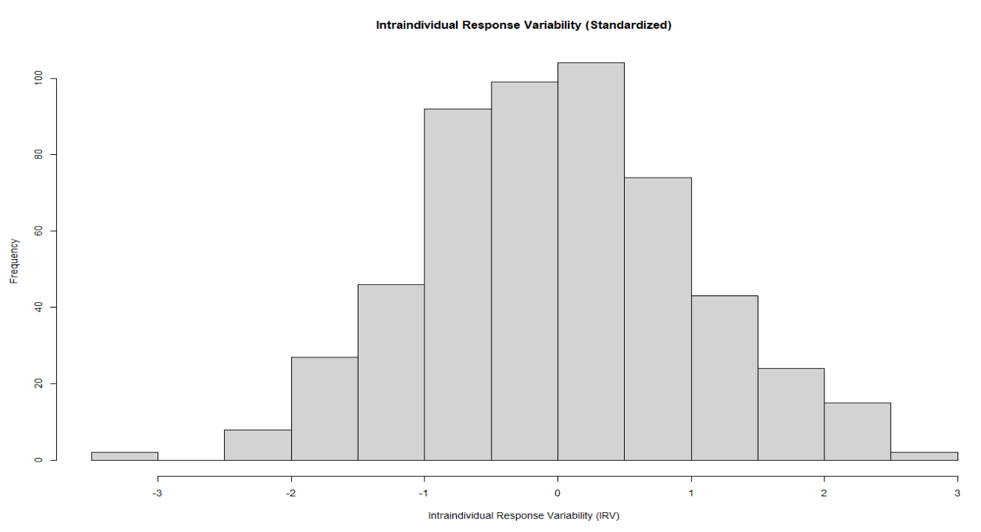


Figure S1. Histogram of Intraindividual Response Variability (Pre-Exclusions)


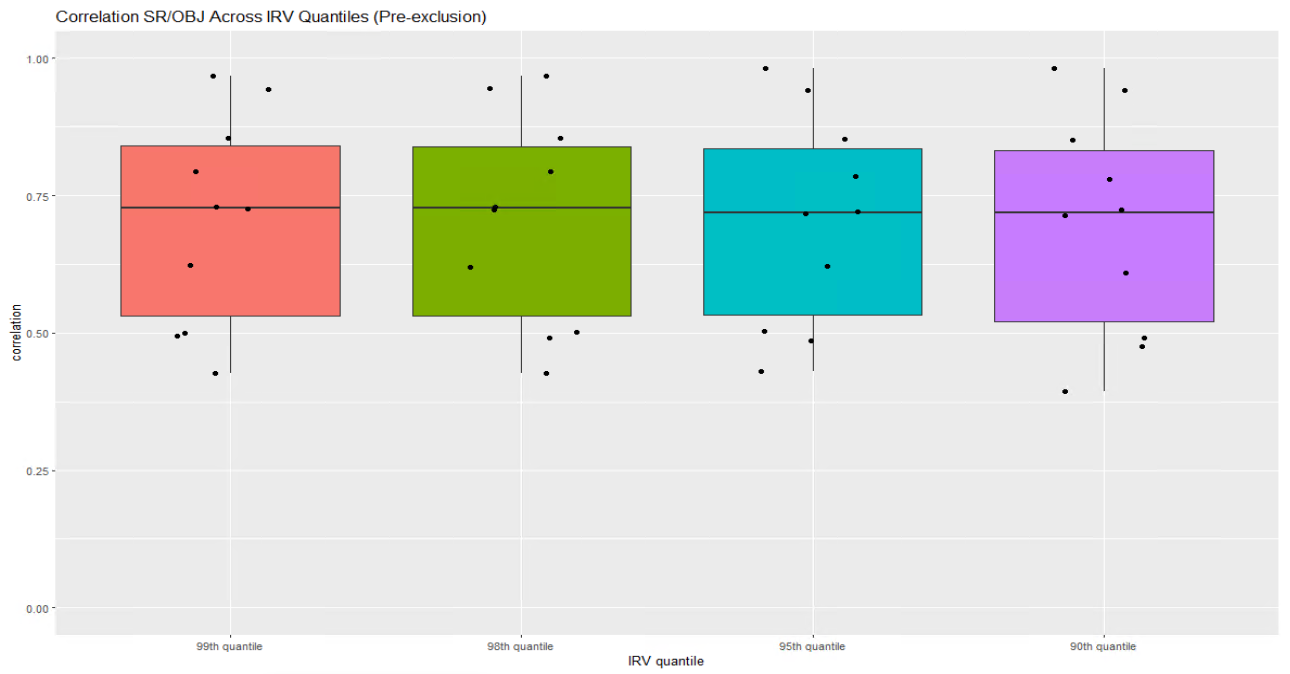


Figure S2. Box Plot of Intraindividual Response Variability (Pre-Exclusions) in the 90-99^th^ percentile.


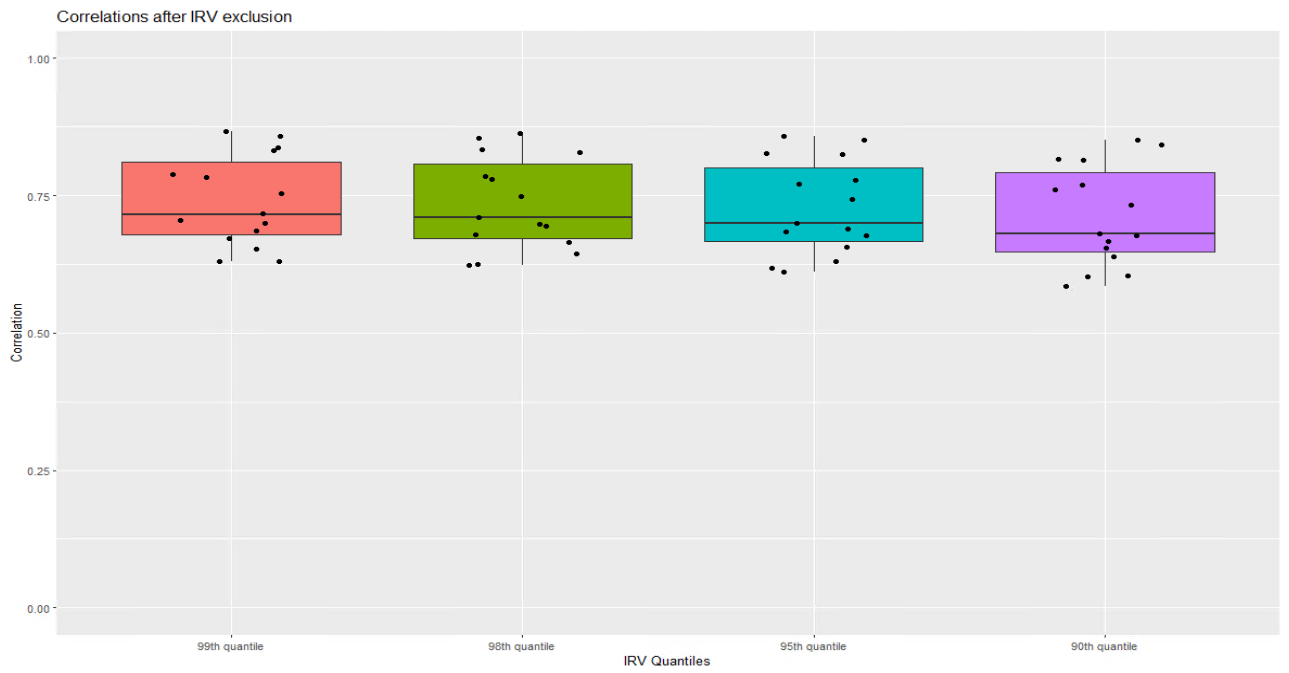


Figure S3. Box Plot of Intraindividual Response Variability (Post-exclusions) in the 90^th^-99^th^ percentile.

**S11.**

Longstring Response Range


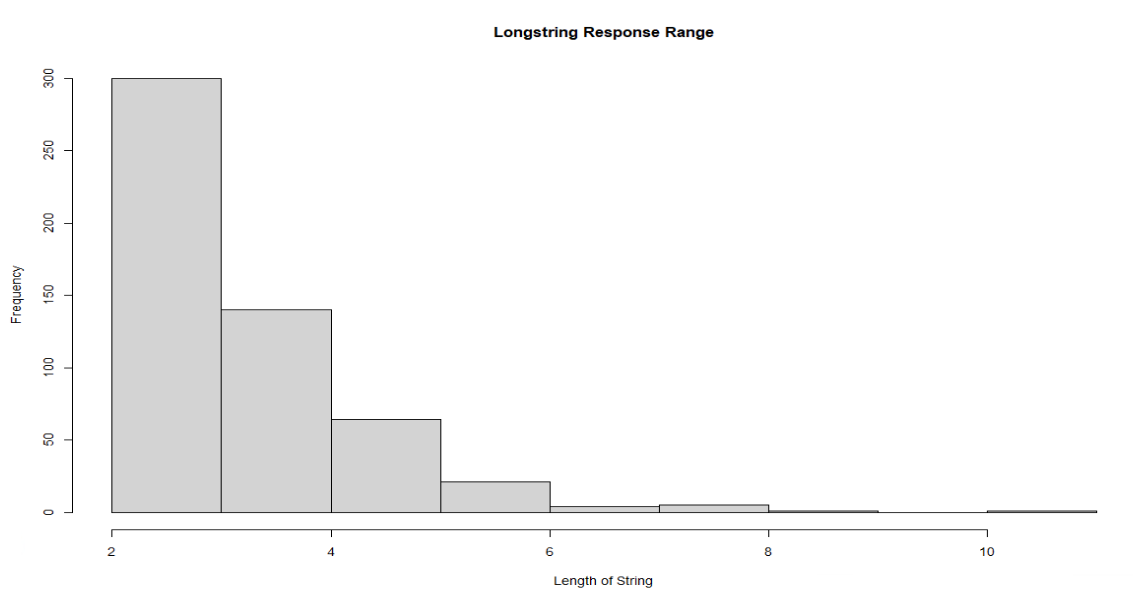


Figure S4. Histogram of Longstring Response Range (Pre-Exclusions).


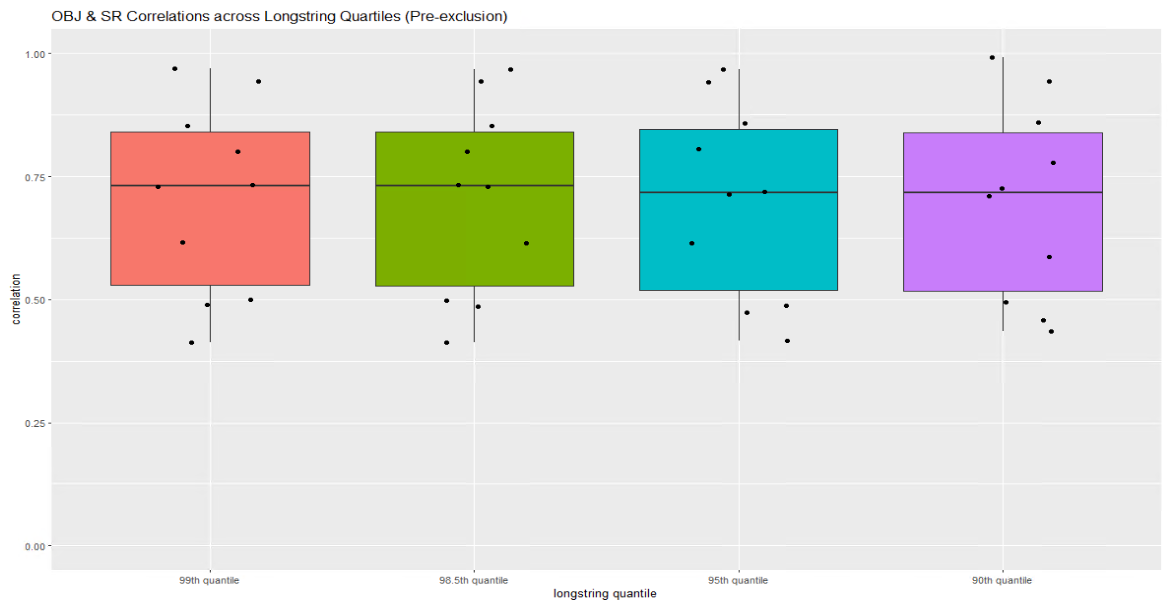


Figure S5. Box Plot of the correlations between objective and subjective Longstring Response Range (Pre-Exclusions).


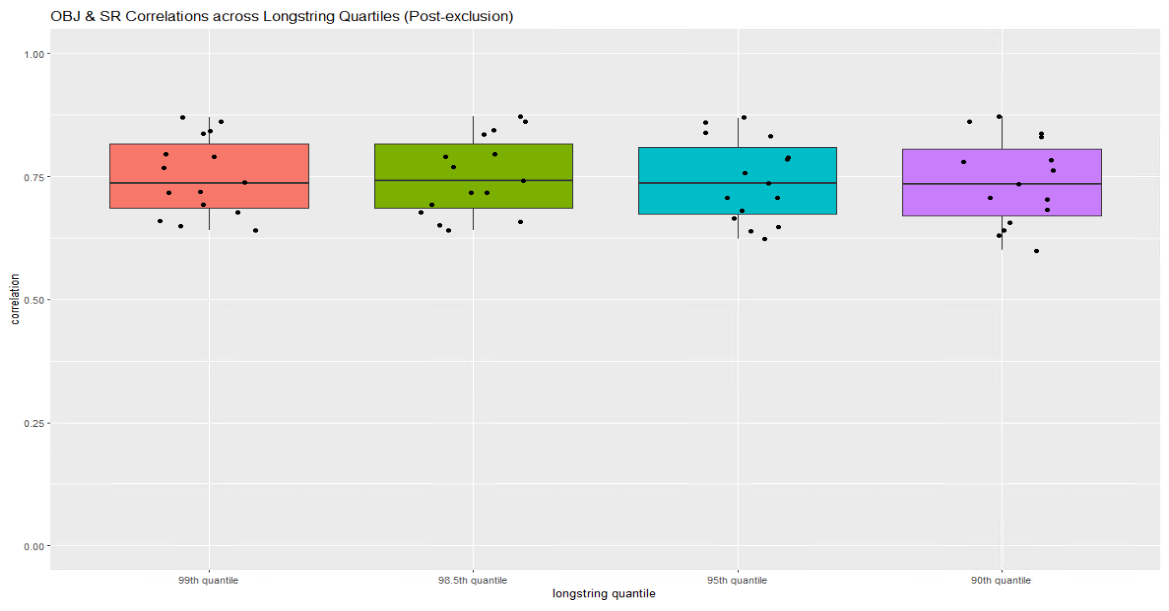


Figure S6.

*Box Plot of the correlation between objective and subjective longstring response range (post-exclusions).*

**S12.**

Inconsistency of Responses


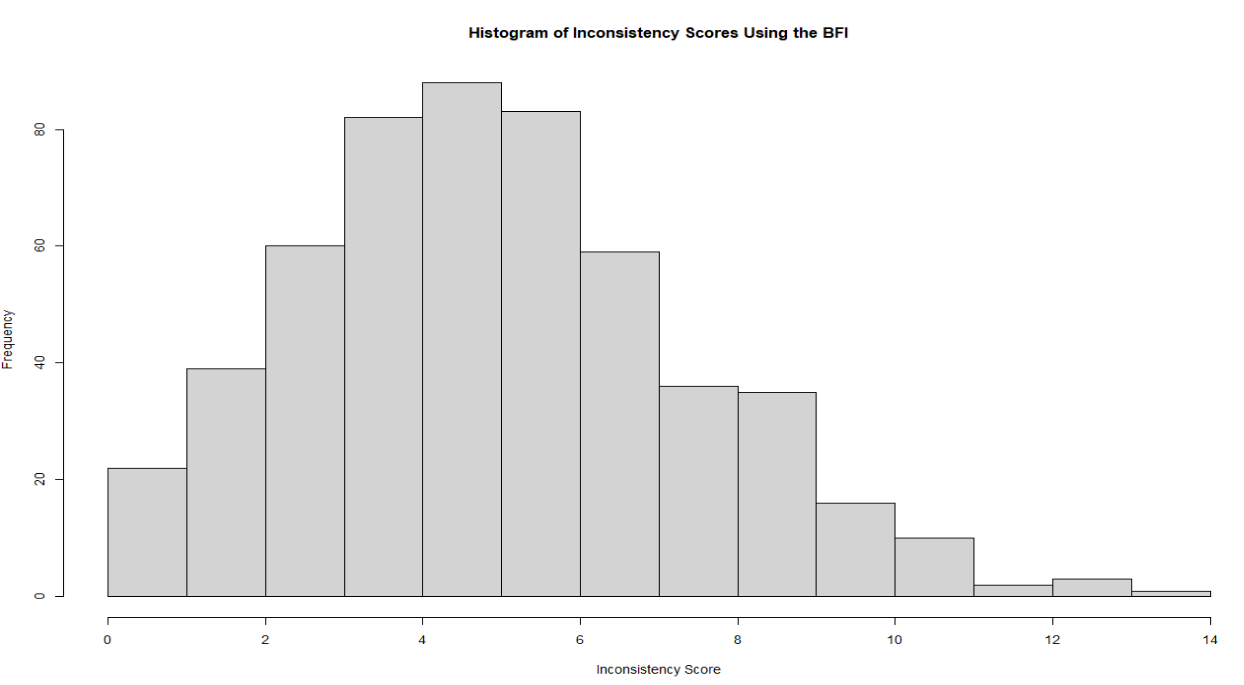


Figure S7. Histogram showing Inconsistency of Responses by Inconsistency Score (Pre-exclusion).


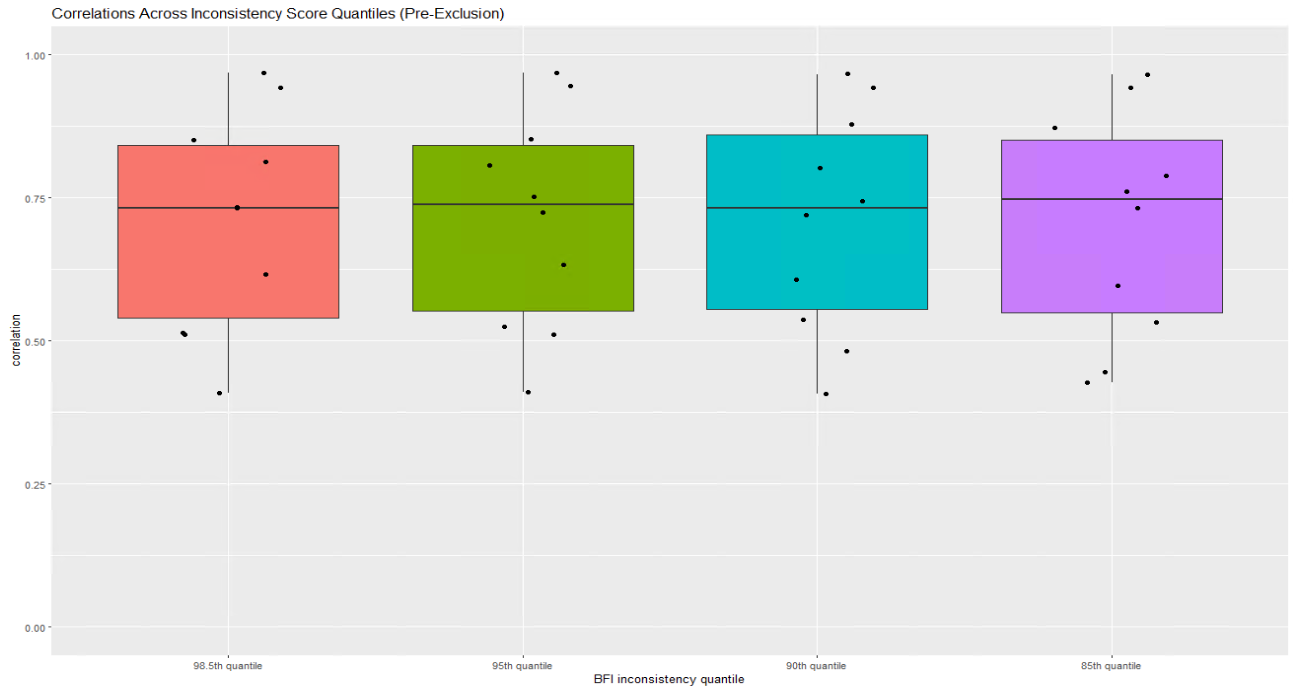


Figure S8. Box plots showing the correlations between objective and subjective Inconsistency of Responses (Pre-exclusion).
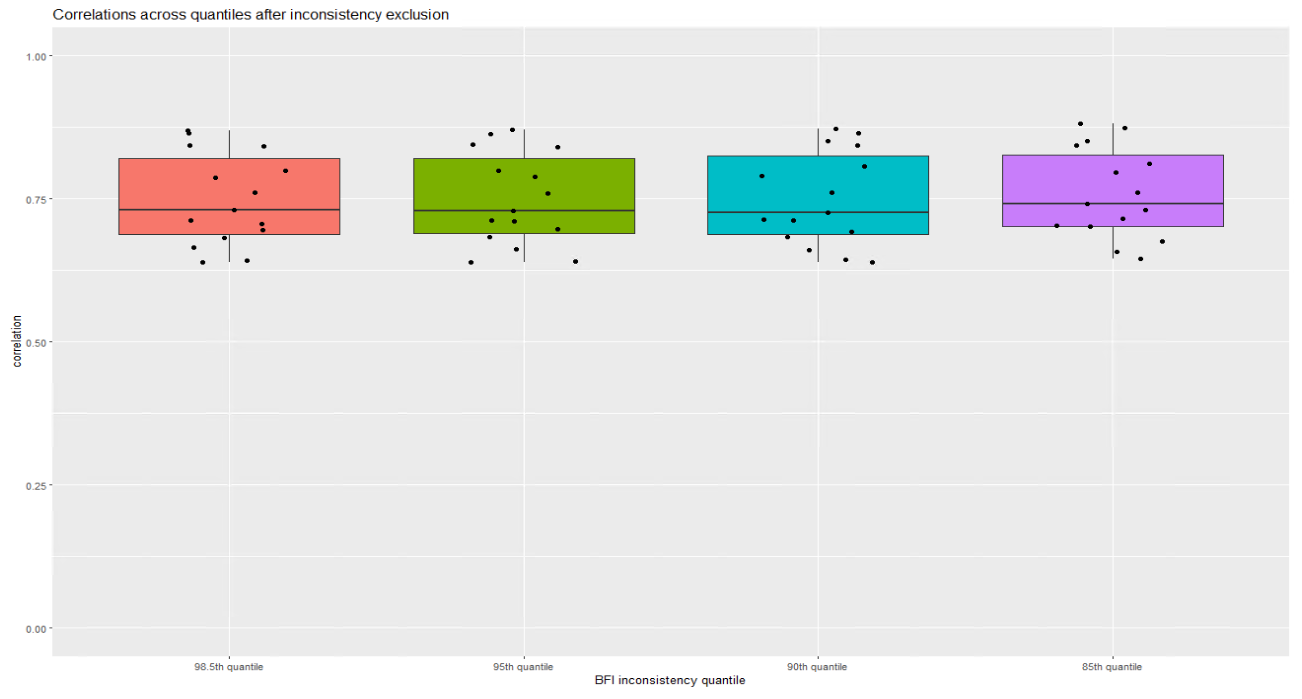


Figure S9.

*Box plots showing the correlations between objective and subjective Inconsistency of Responses (Pre-exclusion).*

**S13.**

Distribution of dataset pre and post Mahalanobis exclusions (N = 27)

Figure S13. Comparison of adjusted objective minutes before and after Mahalanobis correction.


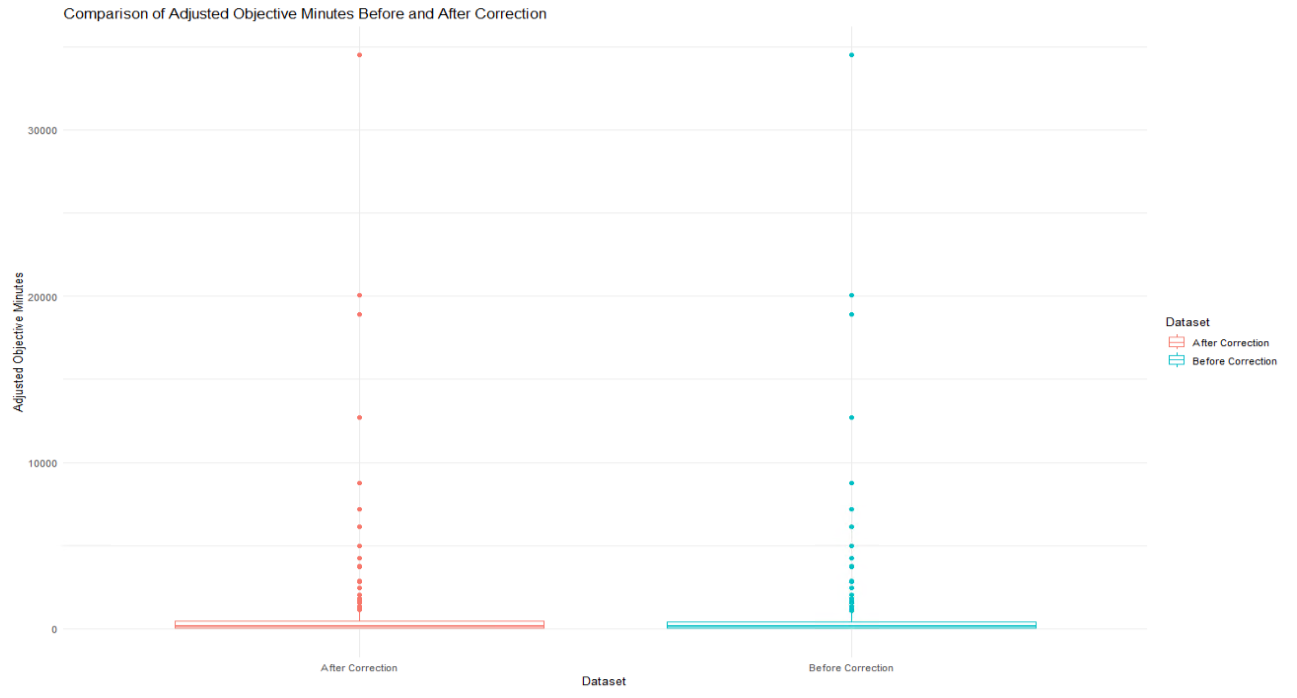


*Note:* Extreme upper outliers are out of scale frame (demonstrating the level of skew).

**S14.**

**Correlation between engagement variables**

Table S3. Spearman’s correlations between self-reported and objective (app-verified) usage.

| Variable | r | df | *p* |
| --- | --- | --- | --- |
| Unadjusted Minutes | 0.513 | 489 | <.001* |
| Winsorised Minutes | 0.768 | 481 | <.001* |
| Unadjusted Sessions | 0.775 | 534 | <.001* |
| Unadjusted Days | 0.495 | 156 | <.001* |
| Unadjusted Streaks | 0.999 | 159 | <.001* |
| Unadjusted Minutes per Session | 0.962 | 327 | <.001* |
| Subset Minutes | 0.631 | 475 | <.001* |

*** = significant after correction for multiple comparisons.

**S15.**

Engagement by App


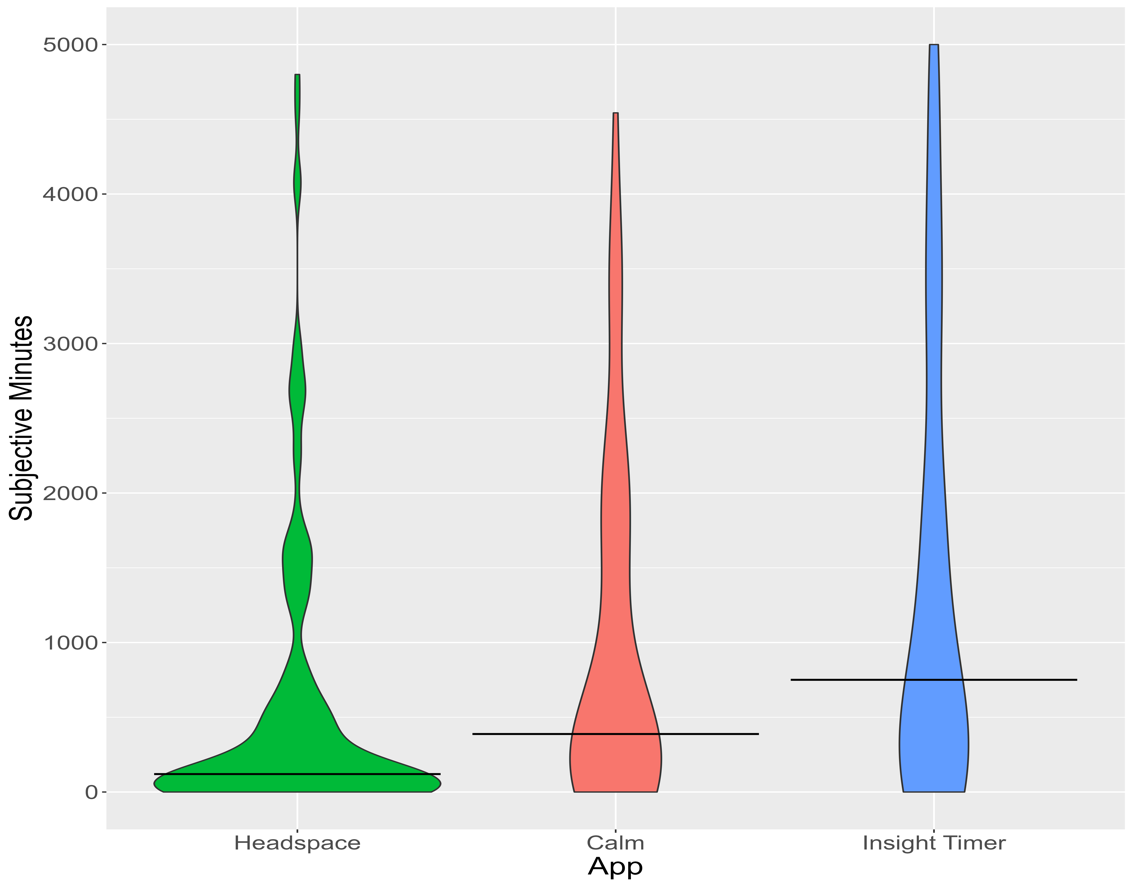


Figure S10. Total subjective minutes reported across the three most popular apps.

*Note***:** The y-axis has been truncated to 5000 minutes but retains 83% of non-finite data.

Users of Headspace engaged less than Calm users (Headspace: *M* = 2016.08, *SD* = 4689.88 Calm: *M* = 6462.52, *SD* = 12889.28). Insight timer users engaged more than Headspace users (*M* = 6681.87, *SD* = 11013.87). There were no significant differences between Insight Timer and Calm.


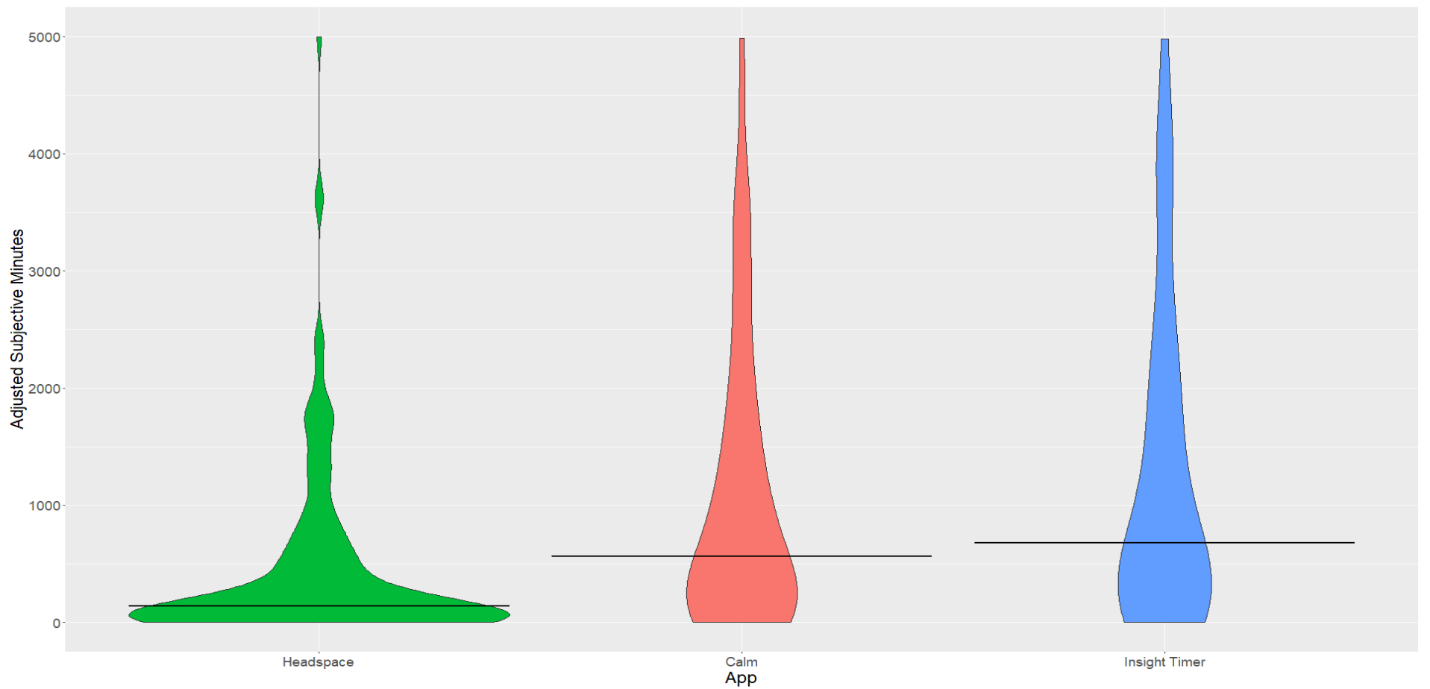


Figure S11. Subjective minutes adjusted for app duration in years by app.

*Note***:** The y-axis has been truncated to 5000 minutes for purposes of visualisation but retains 90% of non-finite data.

Users of Headspace engaged less than Calm users (Headspace: *M* = 1129.02, *SD* = 3400.48, Calm: *M* = 2862.99, *SD* = 4901.78). Insight timer users engaged more than Headspace users (*M* = 2366.35, *SD* = 3555.14). There were no significant differences between Insight Timer and Calm.


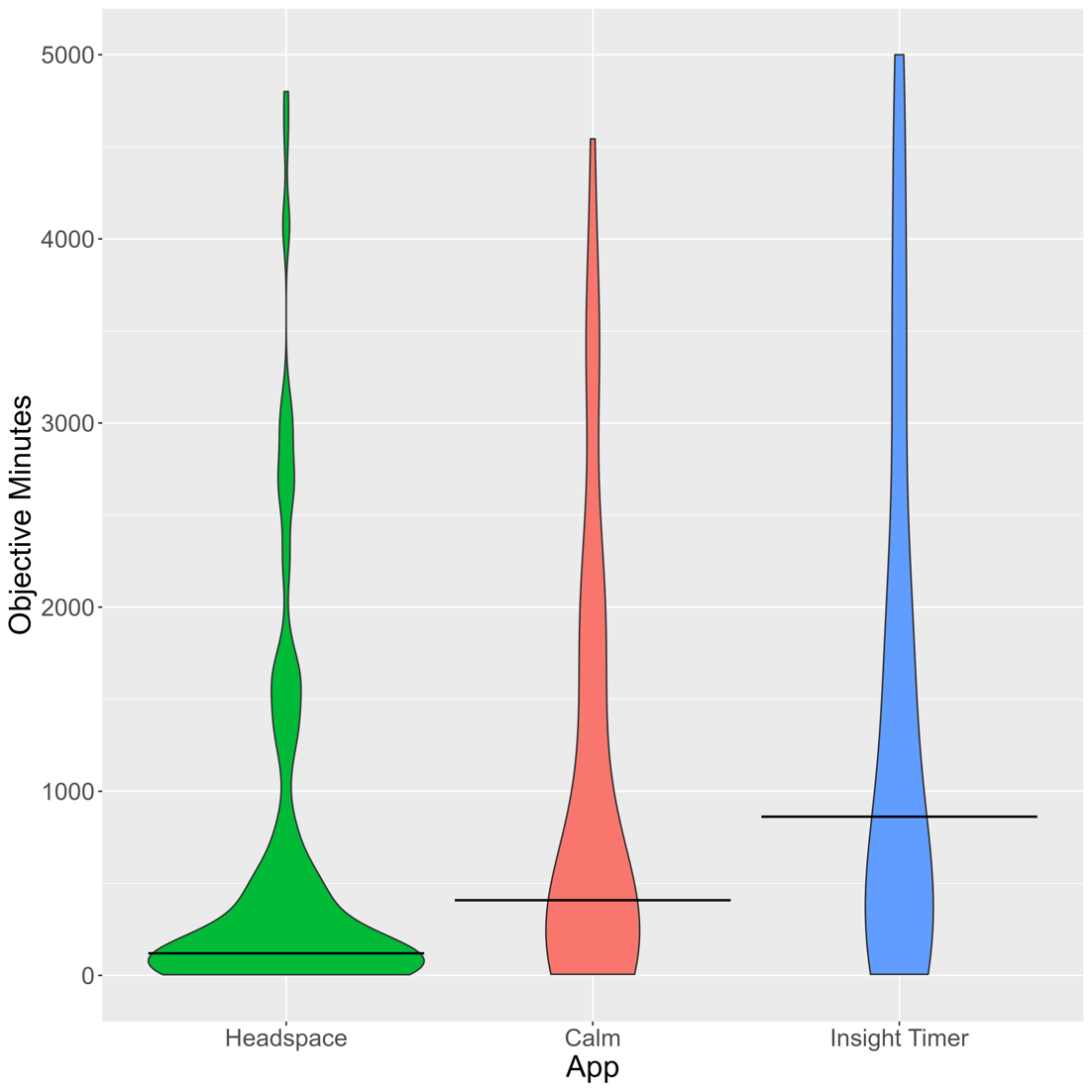


Figure S12. Objective minutes adjusted for app duration in years by app.

*Note***:** The y-axis has been truncated to 5000 minutes for purposes of visualisation but retains 90% of non-finite data.

Headspace: 1097.79 = X, *SD* = 3202.31, Calm: *M* = 2800.14, *SD* = 4917.92. There were no significant differences between the three apps in unadjusted objective minutes.

**S16**

Multivariate Analysis of Variance by App Used.

A Multivariate Analysis of Variance (MANOVA) was conducted to determine the effect of app type on engagement minutes per day. The results indicated a significant effect of app type on engagement, F(2, 682) = 3.64, *p* <.001**.

Table S4. Multivariate Analysis of Variance in engagement (MANOVA) by app used.

|  | ***df*** | ***Value*** | ***F*** | ***Hypothesis df*** | ***Error***  ***df*** | ***P*** |
| --- | --- | --- | --- | --- | --- | --- |
| Pillai’s Trace | 2 | 0.06 | 3.64 | 6 | 682 | .001** |
| Wilk’s Lambda | 2 | 0.93 | 3.67 | 6 | 680 | .001** |
| Hotelling’s trace | 2 | 0.07 | 3.70 | 6 | 678 | .001** |
| Residuals | 342 |  |  |  |  |  |

**S17.**

Regression Models for the four definitions of minutes of engagement.

Coefficients for factors in the adjusted objective minutes model.

Table S5. Regression model for adjusted objective minutes.

| Variable | Estimate (new) | Standard Error (new) | T value  (new) | Pr (>\|t\|)  (new) |
| --- | --- | --- | --- | --- |
| (Intercept) | -0.260*** | 0.005 | -47.199 | <.001*** |
| ***User Factors*** |  |  |  |  |
| Sex | 0.001 | 0.004 | 0.242 | 0.810 |
| Country of Residence (United Kingdom) | <0.001 | 0.004 | 0.186 | 0.853 |
| Age | 0.003 | 0.003 | 1.047 | 0.297 |
| Education | 0.033 | 0.068 | 0.486 | 0.628 |
| BFI Openness | 0.010* | 0.004 | 2.544 | 0.012 |
| Readiness to Change | <0.001 | 0.005 | 0.046 | 0.964 |
| Expectations (Match) | <0.001 | 0.004 | 0.092 | 0.927 |
| Expectations for Sleep | -0.002 | 0.003 | -0.614 | 0.540 |
| Expectations for Stress | <0.001 | 0.005 | 0.144 | 0.885 |
| Expectations for Anxiety | 0.001 | 0.005 | 0.219 | 0.827 |
| Expectations for Happiness | -0.005 | 0.007 | -0.804 | 0.423 |
| Expectations for Thriving | 0.008 | 0.009 | 0.960 | 0.339 |
| Expectations for Performance Enhancement | -0.004 | 0.006 | -0.629 | 0.530 |
| ***App Factors*** |  |  |  |  |
| DWAI (Goal) | -0.009 | 0.010 | -0.852 | 0.396 |
| DWAI (Bond) | -0.004 | 0.005 | -0.865 | 0.389 |
| DWAI (Task) | 0.010 | 0.012 | 0.746 | 0.457 |
| uMARS (Appeal) | -0.012 | 0.008 | -1.565 | 0.120 |
| UMARS (Perceived Quality) | 0.025* | 0.009 | 2.758 | 0.007 |
| uMARS (Perceived Impact) | -0.006 | 0.007 | -0.955 | 0.341 |
| *R2* | 0.158 |  |  |  |

**Notes:**

Adjusted objective minutes: Minutes of use per year adjusted for objective total duration of use in days The table presents the regression coefficients (Estimates), standard errors (Std. Error), t-values (t), and significance levels (p).

The table presents the regression coefficients (Estimates), standard errors (Std. Error), t-values (t), and significance levels (p).

Multiple R-squared; 0.2643, Adjusted R-squared: 0.158

Table S6. Regression model for unadjusted objective minutes

| Variable | Estimate  (new) | Standard Error (new) | T value  (new) | Pr (>\|t\|)  (new) |
| --- | --- | --- | --- | --- |
| (Intercept) | -0.228*** | 0.003 | -57.014 | <.001 |
| ***User Factors*** |  |  |  |  |
| Sex | <0.001 | 0.001 | 0.187 | 0.852 |
| Country of Residence (United Kingdom) | 0.002 | 0.002 | 0.882 | 0.378 |
| Age | -<.001 | 0.002 | -0.254 | 0.799 |
| Education | 0.153*** | 0.036 | 4.214 | <.001 |
| BFI Openness | 0.003^#^ | 0.002 | 1.781 | 0.075 |
| Readiness to Change | 0.005* | 0.002 | 2.480 | 0.013 |
| Expectations (Match) | -0.003 | 0.002 | -1.378 | 0.169 |
| Expectations for Sleep | 0.004^#^ | 0.002 | 1.941 | 0.052 |
| Expectations for Stress | -<.001 | 0.002 | -0.246 | 0.806 |
| Expectations for Anxiety | -0.001 | 0.002 | -0.580 | 0.562 |
| Expectations for Happiness | 0.002 | 0.003 | 0.722 | 0.471 |
| Expectations for Thriving | 0.003 | 0.003 | 1.125 | 0.261 |
| Expectations for Performance Enhancement | -0.003 | -0.002 | -1.227 | 0.220 |
| ***App Factors*** |  |  |  |  |
| DWAI (Goal) | 0.004 | 0.004 | 1.126 | 0.261 |
| DWAI (Bond) | -0.003 | 0.002 | -1.526 | 0.128 |
| DWAI (Task) | 0.002 | <.001 | 0.771 | 0.441 |
| uMARS (Appeal) | <.001 | 0.002 | 0.029 | 0.977 |
| uMARS (Perceived Quality) | 0.003** | 0.003 | 2.679 | 0.008 |
| uMARS (Perceived Impact) | -<.001 | 0.002 | -0.022 | 0.982 |
| *R^2^* | 0.150 |  |  |  |
|  |  |  |  |  |

**Notes:**

***p < .001, **p < .01, *p < .05.

Unadjusted Objective minutes: total minutes of app-verified use.

Multiple R-squared: 0.184, Adjusted R-squared: 0.150

Table S7. Regression model for adjusted self-reported minutes

| Variable | Estimate (new) | Standard Error (new) | T value  (new) | Pr (>\|t\|)  (new) |
| --- | --- | --- | --- | --- |
| (Intercept) | -0.355*** | 0.014 | -24.987 | <.001*** |
| ***User Factors*** |  |  |  |  |
| Sex | -0.008 | 0.009 | -0.982 | 0.327 |
| Country of Residence (United Kingdom) | 0.008 | 0.009 | 0.937 | 0.349 |
| Age | 0.004 | 0.008 | 0.465 | 0.642 |
| Education | 0.532*** | 0.155 | 3.437 | <.001 |
| BFI Openness | 0.005 | 0.009 | 0.626 | 0.532 |
| Readiness to Change | 0.027** | 0.009 | 2.923 | 0.004 |
| Expectations (Match) | 0.023* | 0.011 | 2.148 | 0.032 |
| Expectations for Sleep | -0.008 | 0.009 | -0.892 | 0.373 |
| Expectations for Stress | 0.012 | 0.013 | 1.022 | 0.307 |
| Expectations for Anxiety | -0.020^#^ | 0.013 | -1.670 | 0.095 |
| Expectations for Happiness | 0.009 | 0.012 | 0.741 | 0.459 |
| Expectations for Thriving | -0.006 | 0.012 | -0.349 | 0.727 |
| Expectations for Performance Enhancement | -0.015 | 0.011 | -1.327 | 0.185 |
| ***App Factors*** |  |  |  |  |
| DWAI (Goal) | -0.020 | 0.0178 | -1.147 | 0.252 |
| DWAI (Bond) | -0.013 | 0.013 | -1.016 | 0.310 |
| DWAI (Task) | 0.024 | 0.020 | 1.211 | 0.227 |
| uMARS (Appeal) | -0.016 | 0.011 | -1.449 | 0.148 |
| uMaARS (Perceived Quality) | 0.041*** | 0.012 | 3.374 | <.001 |
| uMARS (Perceived Impact) | -0.003 | 0.011 | -0.250 | 0.802 |
| *R^2^* | 0.126 |  |  |  |

Notes:

*** = p < .001, ** = p < .01, * = p < .05.

Adjusted objective minutes: Self-reported minutes of use per year adjusted for total self-reported duration of use in days/years.

The table presents the regression coefficients (Estimates), standard errors (Std. Error), t-values (t), and significance levels (p).

Multiple R-squared: 0.162, Adjusted R-squared: 0.128

Table S8. Regression Model for unadjusted self-report minutes

| Variable | Estimate (new) | Standard Error (new) | T value  (new) | Pr (>\|t\|)  (new) |
| --- | --- | --- | --- | --- |
| (Intercept) | -0.371*** | 0.007 | -52.275 | <.001 |
| ***User Factors*** |  |  |  |  |
| Sex | 0.002 | 0.003 | 0.632 | 0.528 |
| Country of Residence (United Kingdom) | 0.004 | 0.004 | 1.055 | 0.292 |
| Age | -0.001 | 0.003 | -0.396 | 0.692 |
| Education | 0.237*** | 0.066 | 3.582 | <.001 |
| BFI Openness | 0.006^#^ | 0.003 | 1.783 | 0.075 |
| Readiness to Change | 0.008* | 0.003 | 2.419 | 0.015 |
| Expectations (Match) | -0.004 | 0.003 | -1.306 | 0.192 |
| Expectations for Sleep | 0.009* | 0.004 | 2.26 | 0.024 |
| Expectations for Stress | <.001 | 0.005 | 0.142 | 0.887 |
| Expectations for Anxiety | -0.003 | 0.004 | -0.797 | 0.426 |
| Expectations for Happiness | <.001 | 0.005 | 0.185 | 0.853 |
| Expectations for Thriving | 0.006 | 0.005 | 1.11 | 0.267 |
| Expectations for Performance Enhancement | -0.003 | 0.004 | -0.776 | 0.438 |
| ***App Factors*** |  |  |  |  |
| DWAI (Goal) | 0.008 | 0.008 | 1.017 | 0.310 |
| DWAI (Bond) | -0.002 | 0.004 | -0.573 | 0.567 |
| DWAI (Task) | 0.004 | 0.006 | 0.549 | 0.583 |
| uMARS (Appeal) | 0.005 | 0.004 | 1.323 | 0.186 |
| uMARS (Perceived Quality) | 0.010* | 0.005 | 2.240 | 0.026 |
| uMARS (Perceived Impact) | 0.002 | 0.004 | 0.420 | 0.674 |
| *R^2^* | 0.137 |  |  |  |

**Notes:**

**p < .001, *p < .01, p < .05.

Unadjusted self-report minutes: total self-reported minutes before adjustment for duration of app use.

The table presents the regression coefficients (Estimates), standard errors (Std. Error), t-values (t), and significance levels (p).

Multiple R-squared: 0.1700, Adjusted R-squared: 0.137

**S18**

Table S9. Confidence Intervals for the correlations between factors and the four main outcome variables

| **Objective Minutes** | | | **Self-report Minutes** | | **Adjusted Objective** | | **Adjusted Self-report** | |
| --- | --- | --- | --- | --- | --- | --- | --- | --- |
| **Predictor** | **2.5 %** | **97.5 %** | **2.5 %** | **97.5 %** | **2.5 %** | **97.5 %** | **2.5%** | **97.5 %** |
| (Intercept) | -0.236 | -0.220 | -0.384 | -0.358 | -0.272 | -0.250 | -0.384654 | -0.329483242 |
| Age | -0.0034877310 | 0.0032998691 | -0.0056271100 | 0.0048764357 | -0.002739136 | 0.009786580 | -0.011067295 | 0.019315577 |
| Sex | -0.0030259440 | 0.0036057640 | -0.0038833300 | 0.0075117772 | -0.4295879 | -0.1363212 | -0.025571382 | 0.008599105 |
| Country | -0.0069177930 | 0.0009849109 | -0.0123751000 | 0.0009830505 | -0.010901870 | 0.005462061 | -0.020253615 | 0.014207039 |
| Readiness to Change | 0.0008139518 | 0.0089622235 | 0.0011100480 | 0.0142606110 | -0.011125058 | 0.011250872 | 0.008216919 | 0.044390479 |
| Matches Expectations | -0.0063163680 | 0.0012780650 | -0.0108636400 | 0.0022560075 | -0.007767788 | 0.008258246 | 0.002036488 | 0.043383622 |
| Expectations for Sleep | 0.0000653378 | 0.0089086932 | 0.0013196480 | 0.0169088919 | -0.008459295 | 0.004397098 | -0.027411724 | 0.009889791 |
| Expectations for Stress | -0.0058352880 | 0.0040316703 | -0.0085656300 | 0.0089470738 | -0.011030658 | 0.012092769 | -0.011822765 | 0.037096527 |
| Expectations for Anxiety | -0.0056788620 | 0.0034573918 | -0.0106525000 | 0.0050278828 | -0.008025288 | 0.011372314 | -0.043125601 | 0.003383295 |
| Expectations for Happiness | -0.0034159030 | 0.0078514110 | -0.0085719420 | 0.0115363651 | -0.018828581 | 0.007128704 | -0.013329873 | 0.032742741 |
| Expectations for Thriving | -0.0029477410 | 0.0089859765 | -0.0051009860 | 0.0147499185 | -0.009703758 | 0.024612624 | -0.037155472 | 0.023436563 |
| Expectations for Performance Enhancement | -0.0075827580 | 0.0019725381 | -0.0104760500 | 0.0051474773 | -0.016245687 | 0.009022925 | -0.035597774 | 0.007635001 |
| Appeal | -0.0040685630 | 0.0040267921 | -0.0023541710 | 0.0111465488 | -0.027602307 | 0.003057529 | -0.038420843 | 0.005360700 |
| DWAI Total | -0.0036632200 | 0.0124745677 | -0.0073780640 | 0.0225779374 | -0.030220961 | 0.010777446 | -0.053023850 | 0.015929448 |
| Perceived Quality | -0.0084553060 | 0.0008564235 | -0.0108683900 | 0.0054187208 | -0.015337190 | 0.004809110 | -0.035467986 | 0.012319191 |
| Perceived Impact | -0.0045694360 | 0.0090859343 | -0.0096385210 | 0.0151254732 | -0.014187202 | 0.034727664 | -0.016037359 | 0.059849673 |
| Education | 0.0021394700 | 0.0142801367 | 0.0017428250 | 0.0201608817 | 0.007055236 | 0.042284974 | 0.017607052 | 0.066756419 |
| Openness | -0.0056889360 | 0.0047070339 | -0.0075856240 | 0.0097035371 | -0.019403335 | 0.006239299 | -0.023722783 | 0.019748776 |
|  | 0.0793018900 | 0.2188293982 | 0.1047197000 | 0.3577394007 | -0.104938913 | 0.156179059 | 0.225811943 | 0.830657114 |
|  | 0.0000314266 | 0.0073685770 | 0.0000309296 | 0.0132885820 | 0.002728298 | 0.018119349 | -0.013145038 | 0.021129089 |
